# Robust *gdf9* and *bmp15* expression in the oocytes of ovotestes through the Figla-independent pathway in the hermaphroditic black porgy, *Acanthopagrus schlegelii*

**DOI:** 10.1371/journal.pone.0186991

**Published:** 2017-10-26

**Authors:** Guan-Chung Wu, Jia-Wun Luo, Hau-Wen Li, Chen-Hsiu Huang, Ching-Fong Chang

**Affiliations:** 1 Department of Aquaculture, National Taiwan Ocean University, Keelung, Taiwan; 2 Center of Excellence for the Ocean, National Taiwan Ocean University, Keelung, Taiwan; National Cheng Kung University, TAIWAN

## Abstract

More than 1,500 fish species are hermaphroditic, but no hermaphroditic lineage appears to be evolutionarily ancient in fishes. Thus, whether more than one sex at a time was present during the evolutionary shift from gonochorism to hermaphroditism in fishes is an intriguing question. Ectopic oocytes were created in the ovotestes of protandrous black porgy via the withdrawal of estradiol (E2) administration. These ectopic oocytes reprogrammed the surrounding cells, which changed from Sertoli cells to follicle-like cells. We observed that *gdf9* and *bmp15* expression was localized in the primary oocytes and gradually decreased after oocytes entered a secondary oocyte stage. Robust expression of *gdf9* and *bmp15* in ectopic oocytes was associated with the surrounding Sertoli cells. However, blocking Cyp19a1a activity and increasing androgen levels did not stimulate the expression of *gdf9* and *bmp15*. Thus, the robust *gdf9* and *bmp15* expression was not related to the inappropriate male microenvironment. Furthermore, in vitro data demonstrated that *gdf9* and *bmp15* were not downstream genes of Figla signaling. Therefore, our results suggest that there are two independent mechanisms, a Figla-dependent pathway and a Figla-independent pathway, by which oocyte-surrounding cells are altered from a male somatic fate to a female somatic fate. This functional switch might clarify how oocytes created an appropriate microenvironment during the transition from the ancient gonochorism to the present hermaphroditism.

## Introduction

Most hermaphroditic fishes change sex during their lifetime in response to internal or environmental cues. These sequential sex changes in fishes include 3 primary forms: protogyny (female-to-male sex change), protandry (male-to-female sex change), and bi-directional sex change. However, no hermaphroditic lineage appears to be evolutionarily ancient in fishes [1]. Thus, an intriguing question is how the simultaneous presence of more than one sex occurred during the evolutionary shift from gonochorism to hermaphroditism in fish. Another interesting question is why endocrine disrupting compound (EDC)-induced oocytes often survive in ovotestes after fish are transferred to an EDC-free environment.

Unlike gonadal cell sexual fate in mammals, which show low sensitivity for sex steroids, plasma sex steroid levels are important for gonadal differentiation in fish [2, 3]. Plasma sex steroid levels are also important for sex changes in hermaphroditic fish [3–5]. Intersex (ovotestis) in gonochoristic fish is often considered a signature effect of exposure to EDCs, the most common being estrogenic chemicals [6]. Furthermore, the inhibition of aromatase activity by the aromatase inhibitor (AI) results in the male phenotype in gonochoristic fish and female-to-male sex change in hermaphroditic fish [2, 3, 7]. However, AI does not block oocyte formation in medaka (*Oryzias latipes*) [8] or protandrous black porgy [9, 10] during early gonadal development. These data indicate that gonadal germ cells can differentiate to female under very low estradiol (E2) levels. Thus, oocyte formation is not only induced in an E2-dependent manner but also guided in an E2-independent manner.

Phenotypic males are often observed among germ cell-depleted zebrafish (*Danio rerio*) [11, 12] and medaka [13]. These data indicate that the sexual fate of somatic cells is likely regulated by the presence or absence of germ cells. Furthermore, in protogynous wrasse (*Halichoeres trimaculatus*) [14] and grouper (*Epinephelus coioides*) [15], oocyte-depleted follicle cells alter the sexual fate from female soma to male soma during female-to-male sex change. In protandrous black porgy, ectopic oocytes in E2-induced ovotestis mediate reprogramming of the surrounding cells from male to female soma during oocyte growth [9]. These data demonstrate that sexual development in somatic cells is quite labile and plastic. Therefore, these data also suggest that oocytes may regulate the sexual differentiation of soma.

Growth differentiation factor 9 (*GDF9*) and bone morphogenetic protein 15 (*BMP15*) are oocyte-specific growth factors that have been shown to be essential for primordial follicular development and follicle growth in mammals [16, 17]. Furthermore, GDF9 and BMP15 synergistically interact in a specific manner that targets the SMAD3 pathway and that is dependent on ERK1/2 and SRC kinase signaling [18]. Similar to the case in mammals, both *gdf9* and *bmp15* are highly expressed in the ovary in rare minnow (*Gobiocypris rarus*) [19], European sea bass (*Dicentrarchus labrax*) [20, 21] and Gibel carp (*Carassius auratus gibelio*) [22, 23]. In zebrafish, *gdf9* and *bmp15* are expressed in the ovary and testis [24, 25]. Furthermore, *gdf9*/Gdf9 and *bmp15*/Bmp15 show oocyte-specific expression and are highly expressed at the primary oocyte stage in the ovary in Gibel carp [23] and European sea bass [20, 21]. However, in zebrafish, *gdf9* expression was found to be the highest in primary oocytes [25], whereas *bmp15* is expressed at a consistent level at all stages of oocyte development [24]. These data indicate that both *gdf9* and *bmp15* have conserved functions in ovary but not in testis. Furthermore, in zebrafish, hCG (human chorionic gonadotropin) treatment produces stage-dependent inhibition, with the strongest inhibition observed for fully grown follicles and no effect on follicles during primary growth, and ovarian *gdf9* expression is downregulated by hCG [25]. Conversely, the blockage of Bmp15 by Bmp15 antiserum significantly increases oocyte maturation through suppressing the sensitivity of follicles to maturation-inducing hormone (Mih) but not to hCG [24–27]. These data demonstrate that in some fishes, both *gdf9* and *bmp15* are important not only for early follicle development but also for preventing oocyte maturation.

**Fig 1:**
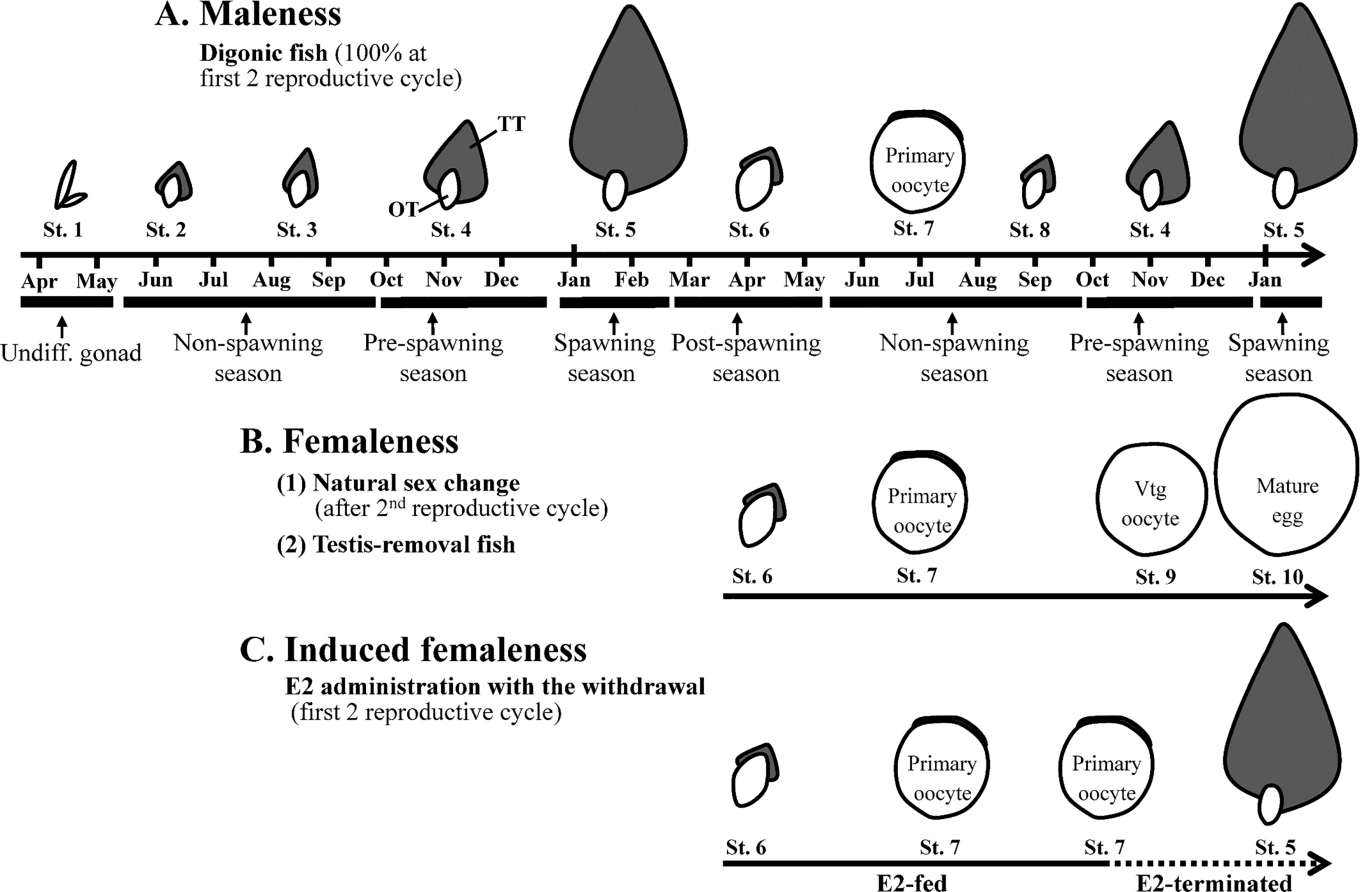
Profiles of gonadal development in 3 different sexual phases. A) Maleness: The fish are functional males in the first two spawning seasons, and then half of them change sex to female function in the third spawning season. Digonic gonads (testis and ovary in one gonad) are separated by connective tissue. The testis exists at various stages of the reproductive cycle until the fish changes to femaleness. Only primary oocytes are found before the sex change occurs. B) Active femaleness: The fish are functional females after sex change or induction by removal of the testis of the digonic gonad. C) Passive femaleness: Long-term estradiol (E2) administration results in a dominant ovary with the primary oocytes in the ovary and a regressed testis in < 2-yr-old fish, and no vitellogenic oocytes are observed in E2-induced sex change fish. This sex change is reversed after E2 administration is withdrawn. The gonadal development is classified into 10 different gonadal stages: undifferentiated gonad (stage 1), differentiated gonad (stage 2), developed testes with spermatogonia (stage 3), developed testes with spermatocytes (stage 4), developed testes with spermatozoa (stage 5), regressing testes with an active ovary (stage 6), dominant ovary with regressed testes (stage 7), regressing ovaries with active testes (stage 8), dominant ovary and oocytes with vitellogenesis (stage 9), and female function with rudimentary testes (stage 10). St. = gonadal stage, Vtg oocyte = vitellogenic oocyte, TT = testicular tissue, OT = ovarian tissue, undiff. = undifferentiated gonad, diff. = differentiated gonad.

To study questions regarding *gdf9* and *bmp15* in the early phase of ovotestis formation during hermaphroditic evolution, we selected protandrous black porgy (*Acanthopagrus schlegelii*) as our experimental animal. Three sexual phases are demonstrated in black porgy: maleness, femaleness, and induced femaleness (Fig 1). This digonic fish has testicular and ovarian tissues separated by connective tissue, and it undergoes a stable sex change: all fish have male function in the first two spawning seasons (maleness, Fig 1A), and then half the fish change sex in the third spawning season (femaleness, Fig 1B) [28]. In addition, reversible sex change can occur in E2-induced female fish < 2 yr old, which can switch from a dominant ovary with a regressed testis to a dominant testis with a regressed ovary (under low E2 levels) after the withdrawal of E2 administration (induced femaleness, Fig 1C) [7, 10]. Thus, we used this property to generate an ovotestis in the testicular tissue [9, 29], allowing us to investigate how ectopic oocytes in an ovotestis could reprogram the surrounding cells from male fate to female fate. Our studies aimed to explain why both sexes can simultaneously exist in the gonad and produce an ovotestis under low plasma E2 levels during the transition from gonochorism to hermaphroditism.

Here, our experimental data indicated that *gdf9* and *bmp15* were most highly expressed in primary oocytes. Thus, the presence of robust *gdf9* and *bmp15* expression in the ectopic oocytes in ovotestes may help the oocytes create an appropriate microenvironment through reprogramming the surrounding cells from Sertoli cells to follicle-like cells. In addition, our *in vitro* data also indicated that the presence of robust *gdf9* and *bmp15* expression in ectopic oocytes did not correlate with the oocyte-specific gene *factors in germline alpha* (*figla*), whose expression is tightly related to the surrounding cell type: high *figla* expression when surrounded by Sertoli cells and low *figla* expression with follicle-like cells. Taken together, follicular formation (reprogramming surrounding cells from Sertoli cells to follicle-like cells) in ectopic oocytes is not regulated solely in a Figla-dependent manner. This study provides a novel hypothesis for the transition from gonochorism to hermaphroditism: a weak male environment results in oocyte differentiation, and then oocytes can survive and reprogram the surrounding cells.

## Materials and methods

### Animals and experimental design

The experimental fish were acclimated in seawater to the pond environment at the National Taiwan Ocean University culture station with a natural lighting system. All procedures and investigations were approved by the National Taiwan Ocean University Institutional Animal Care and Use Committee (approved number 104007) and were performed in accordance with standard guiding principles. The fish were anesthetized in 2-phenoxyethanol (0.5 ml/l water) during sampling.

#### Experiment 1

The expression profiles of *gdf9* and *bmp15* during gonadal development and sex change. Fish were collected during maleness (< 2 yr old) and femaleness for genetic analysis and *in situ* hybridization. To examine ovarian growth in femaleness, we surgically removed the testicular part of the digonic gonad at stage 6 (regressing testes with an active ovary). Surgery was performed as described previously [10, 29]. All fish survived. Gonad samples were collected at 2-mo intervals (n = 6–8) for histology and genetic analysis.

#### Experiment 2

The characteristics of ectopically located oocytes in E2-induced ovotestes. We created an ovotestis by ectopically generating oocytes in the testicular region as described in our previous studies [9, 29]. Fish (n = 500) were fed a diet containing E2 (6 mg/kg feed; Steraloids, Newport, USA), beginning at stage 1 (undifferentiated gonad; 2-mo-old) and continuing for 4-mo (stage 2, differentiating gonad), after which E2 administration was terminated. Gonad samples were collected monthly (n = 8–30) for histology. All fish survived. An antibody against the Sertoli cell marker Doublesex and mab-3-related transcription factor 1 (Dmrt1) was used to identify the sexual type of cells surrounding ectopic oocytes in the testes.

#### Experiment 3

Effects of sex steroids on *gdf9* and *bmp15* gene expression. To determine the effects of male microenvironment on the expression of *gdf9* and *bmp15*, fish at gonadal stage 7 (dominant ovary with regressed testes) were intraperitoneally (i.p.) injected with aromatase inhibitor (AI, 1,4,6-androstatriene-3,17-dione, 5 mg/kg body weight; Steraloids) and methyltestosterone (MT, 1 mg/kg body weight; Kingyoker, Taipei City, Taiwan) at days 0, 2, and 4 (n = 8). All fish survived. Gonads were collected 1 day after the third injection (day 5), and gene expression was analyzed.

#### Experiment 4

Effects of Figla signaling on *gdf9* and *bmp15* gene expression. Our previous studies showed that prolonged and high Figla expression was only found in ectopic oocytes surrounded by Sertoli cells but not follicle-like cells in the ovotestes [9]. To examine the correlation between Figla signaling and *gdf9* and *bmp15* expression in ectopic oocytes, we developed an *in vitro* oocyte culture system to induce *figla* expression by using expression vectors. The vectors were delivered on day 1 (one day after the isolation of the suspended cells), and then samples were collected 2 days after transfection, and gene expression was analyzed.

#### Cloning of black porgy *gdf9* and *bmp15*

Total RNA in black porgy ovary was extracted using TRIzol reagent (Invitrogen, Carlsbad, CA, USA). Total RNA was reversely transcribed to the first-strand cDNA using Superscript III (Invitrogen) with oligo (dT)12–18 primers (Promega, Madison, WI, USA). To amplify the partial cDNA fragment of black porgy *gdf9* and *bmp15*, PCR primers were designed based on conserved nucleotide sequence of European sea bass and fishes (Table 1). cDNA sequences encoding region of *gdf9* and *bmp15* were elongated by using the SMART 3’-rapid amplification of cDNA ends (RACE) kit (BD Biosciences Clonetech, Mountain View, CA, USA). Primers for RACE were listed in Table 1. The cDNA sequence of *gdf9* (GenBank accession no. KY427738) and *bmp15* (GenBank accession no. KY427737) was used to design the specific primer for RNA analysis and synthesize the RNA probe for *in situ* hybridization.

**Table 1:** Primers used in this study.

Gene	Orientation	Sequence	Analysis
*gdf9*	Sense	5'-GAYTKATMAMRCCRCRRGAYGAGT-3'	partial cDNA
	Antisense	5'-GSACAGAGGAGTCMAGTTST-3'	
*bmp15*	Sense	5'-CTCACCGCCCAGTACTGGT-3'	partial cDNA
	Antisense	5'-GCCGTACAAGTACATGCCCATGAG-3'	
*gdf9*	Sense	5'-CACCTGCCGGAGCTGCTTCCCAGCTCT-3'	3' RACE
	Nested Sense	5'-CACCGAAGTACAACCCCAGGTA-3'	
*bmp15*	Sense	5'-CTGGCGTGCCCAAGAACCGCTGCAAGCT-3'	3' RACE
	Nested Sense	5'-CTGGGGTCACTACTTCATCGCT-3'	
*gdf9*	Sense	5'-GACTGATAAAACGCGAGACGAGT-3'	ISH probe
	Antisense	5'-GGACAGAGGAGTCAAGTTTGT-3'	
*bmp15*	Sense	5'-CTCACCGCCCAGTACTGGT-3'	ISH probe
	Antisense	5'-GCCGTACAAGTACATGCCCATGAG-3'	
*gdf9*	Sense	5'-GACCAGAAGAGCAGAAGGAACTG-3'	qPCR
	Antisense	5'-CATCAAGAGAGGCCGAAGAAA-3'	qPCR
*bmp15*	Sense	5'-GCTACCATTCCCCCAACCA-3'	qPCR
	Antisense	5'-CGCCCAGGTCGTTGATG-3'	qPCR
*dmrt1*	Sense	5'-GGAGGAGCTCGGGATTTGTAGT-3'	qPCR
	Antisense	5'-CAGTCTGCACCAGCTTCATTTT-3'	qPCR
*amh*	Sense	5'-GCCTCACTGTGTCCCTTGAAA-3'	qPCR
	Antisense	5'-ACCAGTGGGACAGGACATGTG-3'	qPCR
*foxl2*	Sense	5'-GAATAAAAAAGGCTGGCAGAACA-3'	qPCR
	Antisense	5'-CCCGCGGAACTTTGATGA-3'	qPCR
*cyp19a1a*	Sense	5'-ACAAACCCGACGAATTCAGACT-3'	qPCR
	Antisense	5'-CCCGAACGGCTGGAAGTA-3'	qPCR
*figla*	Sense	5'-CAGGAACTTGAACACCATGTTCTC-3'	qPCR
	Antisense	5'-CTTACGGTCTGGTCGCATTAGTG-3'	qPCR
*gapdh*	Sense	5'-AGGCTTCCTTAATCTCAGCATAAGAT-3'	qPCR
	Antisense	5'-GGTGCCTGTGGCTGATGTG-3'	qPCR

### Gonadal histology, *in situ* hybridization, immunohistochemical staining and immunofluorescence staining

Hematoxylin-eosin staining, *in situ* hybridization (ISH), immunohistochemical (IHC) staining and immunofluorescence (IF) staining were performed as described previously [10, 30, 31]. Fish gonads were fixed with 4% paraformaldehyde in PBS. cDNA fragments of *gdf9* and *bmp15* were used to synthesize the RNA probe for ISH (Table 1). For ISH, digoxigenin-11-UTP was used to label RNA with digoxigenine (Roche, Penzberg, Germany). Anti-sense digoxigenin-labeled antisense and sense probes were used to detect the localization of *gdf9* and *bmp15*. The probe was developed overnight, using the standard protocol in our laboratory. Immunostaining was performed with preabsorbed alkaline phosphatase-conjugated sheep anti-digoxigenin antibody (Roche, Penzberg, Germany) at room temperature. Finally, the NBT/BCIP Detection System (Sigma-Aldrich, St. Louis, MO, USA) was used to detect *gdf9* and *bmp15* expression. The gonads (5-μm-thickness sections) were treated with HistoVT One (Nacalai Tesque, Kyoto, Japan) to expose the antigens of the target protein. The polyclonal Doublesex and mab-3-related transcription factor 1 (Dmrt1) antiserum was produced in white rabbits immunized against a peptide fragment of black porgy Dmrt1 (CEASSETPNFTVSSIID). The antisera were prepared by Yao-Hong Biotechnology Inc. (New Taipei city, Taiwan). The specificity of the polyclonal anti-Dmrt1 was confirmed by Western blotting described in our previous study [31]. For IHC staining, each section was rehydrated in PBS and incubated with 3% H_2_O_2_ in PBS. The section was then incubated with 5% nonfat milk powder for 30 min with anti-Dmrt1 (1:1500 dilution) overnight at 4°C. This was followed by incubation with an appropriate biotinylated antibody (Vector, Burlingame, CA, USA). Color formation was amplified with an ABC kit (avidin-biotin, Vector; Burlingame, CA, USA) and DAB (3,3’-diaminobenzidine, Sigma). For IF staining, the section was then incubated with 5% nonfat milk powder for 30 min with anti-Factor in germline alpha (Figla; 1:400 dilution, [9]) overnight at 4°C. Alexa Fluor secondary antibodies (Invitrogen) were used. The specificity of anti-Dmrt1 [31] and anti-Figla [9] has been evaluated in our previous studies. All staining was conducted with triplicate sections for each tissue (n = 3~5 fish in each group).

### RNA analysis

Gonads were collected and homogenized in TRIzol reagent (Invitrogen). This homogenate was used for both RNA analysis. The extraction of total RNA and first-strand cDNA was performed according to the manufacturer’s protocol. Total RNA extracted from the gonad of the representative fish was reversely transcribed to the first-strand cDNA using Superscript III (Invitrogen) with oligo (dT)15 primers (Promega). This first-strand cDNA was used for RT-PCR and quantitative real-time PCR analyses (qPCR). The number of PCR cycles was preliminarily tested and was in the range of the linear curve for the relationship between the number of cycles and the amount of PCR product. As an internal control, *glyceraldehyde-3-phosphate dehydrogenase* (*gapdh*; GenBank accession no. DQ399798) was used. Furthermore, Sertoli cells marker (*dmrt1* and *anti-mullerian hormone*, *amh*) [29, 31], follicle cells marker (*forkhead box l2*, *foxl2* and *P450 aronatase gonad form*, *cyp19a1a*) [10], and oocytes marker (*figl*a) [9] were used to analyze the gene expression patters in control fish and E2-treated fish. Specific primers for *gdf9*, *bmp15*, *dmrt1* (GenBank accession no. AY323953), *amh* (GenBank accession no. GU256046), *foxl2* (GenBank accession no. EU496493), *cyp19a1a* (GenBank accession no. AY273211), *figla* (GenBank accession no. EU496494), and *gapdh* are listed in Table 1. Gene quantification of standards, samples, and controls was conducted simultaneously by qPCR (GeneAmp 7500 Sequence Detection System; Applied Biosystems, Foster City, CA, USA) with SYBR green Master Mix (Applied Biosystems, Vilnius, Lithuania). The PCR specificity was confirmed by a single melting curve (at same temperature) in unknown samples and standards. The respective standard curve of log (transcript concentrations) *vs* CT (the calculated fractional cycle number at which the PCR-fluorescence product is detectable above a threshold) was obtained. The values detected from different amounts of plasmid DNA contained the fragment of the target gene (10 times serial dilution) of the representative samples in parallel with the respective standard curve. The correlations of the standard curve for the gene analyses were at least -0.999. The qPCR assay was conducted with duplicate repeats in each sample. All samples were normalized to *gapdh*, and the highest value (control value) of each gene was defined as one. The *gapdh* transcripts were not significantly different between treatments (data not shown).

### In vitro transfection of oocytes

Ovarian cell preparation and oocyte selection were performed as described previously [29]. Ovaries were collected from testis-removal fish (femaleness) at stage 6 (regressing testes with an active ovary), stage 7, and stage 9 (dominant ovary with oocytes with vitellogenesis). The ovaries were dissociated using trypsin (0.36% trypsin, pH 8.2, 5% fetal bovine serum [FBS]) with gentle pipetting. Large clumps and large oocytes were eliminated from the dispersed ovarian cells and tissues through mesh with pore sizes of 70 μm and 40 μm. Filtered ovarian cells (< 40 μm) were seeded in culture medium (L-15 with 5% FBS, 50 U/ml penicillin, and 50 μg/ml streptomycin). Based on the difference in the adhesive ability between somatic cells and oocytes, dispersed cells were incubated in wells overnight and then divided into 2 groups, adhesive cells and suspended cells. The cell types were confirmed based on gene expression of a germ line marker (*vasa*), oocyte marker (*figla*), and follicle cell marker (*foxl2*). The suspension cells were used for vector delivery. The pcDNA3.1(+) vector (Invitrogen) was used for *figla* expression. The control group was infected with a vector only. The vectors were delivered by Escort^tm^ Transfection Reagent (Sigma-Aldrich) at day 1 (one day after suspended cell isolation) and the samples were then collected at day 3. The vector delivery ability for oocytes was confirmed by PCR (forward primer: 5’-TAGTAATCAATTACGGGGTCATTAG-3’; reverse primer: 5’-ACAAACTCCCATTGACGTCA-3’).

### Data analysis

The data were expressed as the mean ± standard deviation (SD). The values were subjected to analyze by one-way ANOVA, followed by a Student-Newman-Keuls multiple test, with *P* < 0.05 indicating a significant difference. Student’s *t*-test was also conducted to determine significant differences (*P* < 0.05) between treatments.

## Results

### Ovarian *gdf9* and *bmp15* were localized in the oocytes

**Fig 2:**
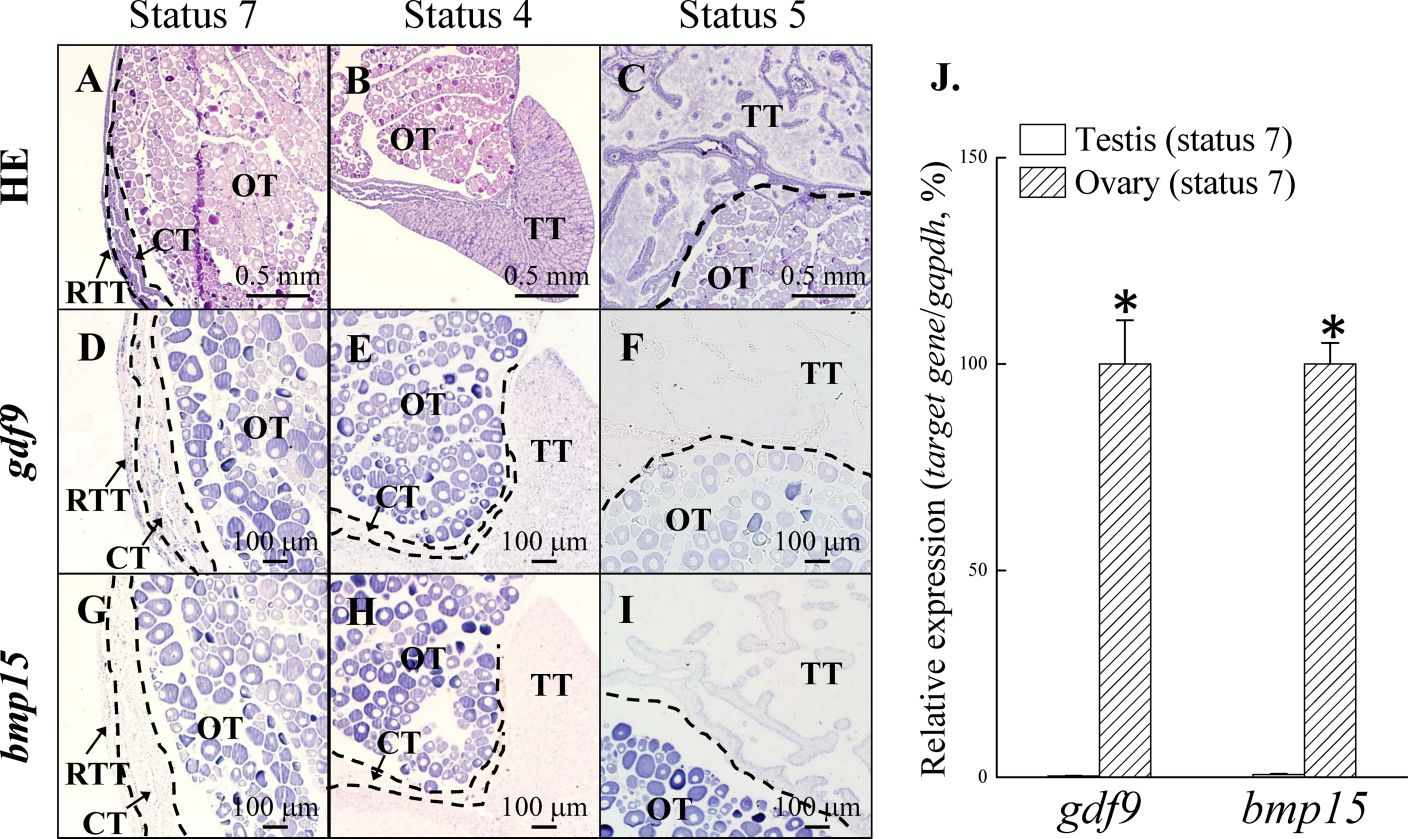
The expression of *gdf9* and *bmp15* in the ovary in the second reproductive cycle. The gonadal stages according to Fig 1. Histological observation of gonads at stage 7 (Fig 1A), stage 4 (Fig 1B), and stage 5 (Fig 1C) was performed. *gdf9* and *bmp15* mRNA expression was detected by *in situ* hybridization (ISH) (D-I) and qPCR (J). Both *gdf9* (D-F) and *bmp15* (G-I) were localized in primary oocytes, and no ISH signal was observed in testes in the second reproductive cycle. qPCR data confirmed that *gdf9* and *bmp15* were expressed at higher levels in the ovary than in the testes (J). An asterisk indicates a significant difference between the testes and ovary (*P* < 0.05). TT, testicular tissue; OT, ovarian tissue; CT, connective tissue; RTT, regressed testicular tissue.

Fig 1 showed a model for gonadal development in the different reproductive cycles (Fig 1A). Histological data (H&E staining) showed that the fish had a digonic gonad (Fig 2A–2C). The ovary was the dominant part of the digonic gonad during the non-spawning season (stage 7; Fig 2A). During the subsequent pre-spawning season, the ovary regressed and the testis grew (stage 4; Fig 2B). During the second spawning season, the testis was the dominant part and functioned as male during the second spawning season (stage 5; Fig 2C). ISH with antisense probes revealed the strong *gdf9* and *bmp15* expression in the ovary compared to the testis (Fig 2D–2I). The expression of *gdf9* and *bmp15* was localized in the primary oocytes (Fig 2D–2I). In the testis, *gdf9* and *bmp15* signals were low or absent at all gonadal stages (stages 4, 5, and 7; from non-spawning season to spawning season) (Fig 2D–2I). No signal was observed from sense probes for *gdf9* (S1A–S1C Fig) and *bmp15* (S1D–S1F Fig). Furthermore, qPCR data confirmed that *gdf9* and *bmp15* were highly expressed in the ovarian tissue compared to the testis tissue in the digonic gonad at stage 7 (Fig 2J).

### Differential expression of *gdf9* and *bmp15* in primary oocytes and vitellogenic oocytes

**Fig 3:**
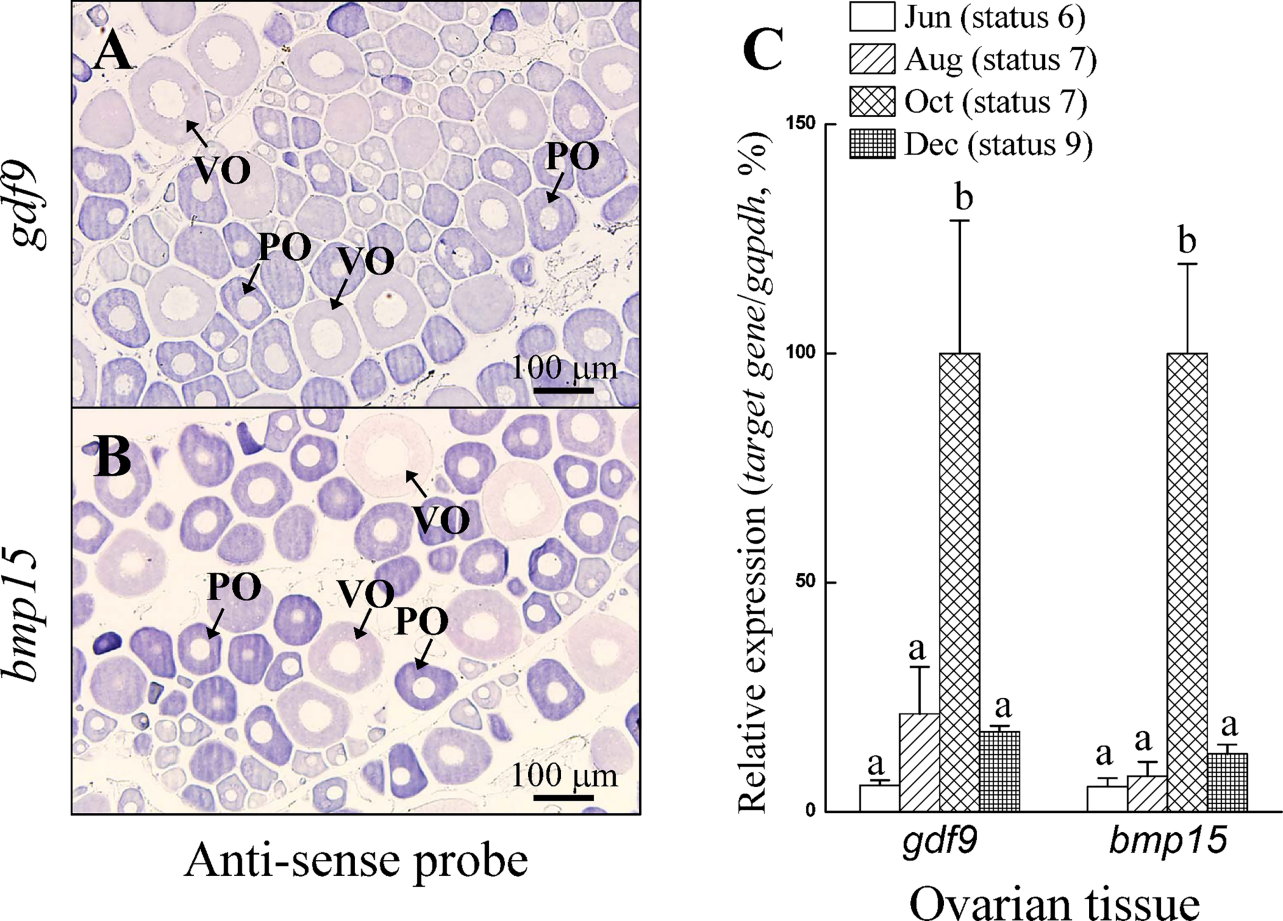
Differential expression of *gdf9* and *bmp15* at the primary oocyte and vitellogenic oocyte stages. *gdf9* and *bmp15* mRNA expression was detected by *in situ* hybridization (ISH) (A and B) and qPCR (C). Expression of *gdf9* was high at the primary oocyte stage and then gradually decreased as oocytes entered the secondary oocyte stage (A). Expression of *gdf9* was slight or absent in the vitellogenic oocyte stage (A). A similar expression pattern was observed for *bmp15* (B). qPCR data confirmed that *gdf9* and *bmp15* were most highly expressed at stage 7 (Oct: ovary with late primary oocytes) compared with the other stages in passive femaleness (C). Different small letters indicate significant differences (*P* < 0.05). PO, primary oocyte; VO, vitellogenic oocyte.

In the third reproductive cycle, half the fish were maintained at maleness (only primary oocytes were observed in the ovary), and the others underwent sex change (entering femaleness) with various stages of oocytes observed in the ovary during pre-spawning season (Fig 1A). ISH staining revealed a slight expression of *gdf9* and *bmp15* in early-stage primary oocytes (small oocyte size), and then *gdf9* and *bmp15* expression gradually increased in late primary oocytes (large oocyte size) (Fig 3A and 3B). In contrast, *gdf9* and *bmp15* expression was low or absent in the vitellogenic oocytes (Fig 3A and 3B). No signal was observed from sense probes for *gdf9* (S1G Fig) and *bmp15* (S1H Fig).

To examine the *gdf9* and *bmp15* expression patterns in the ovary, testis-removal-induced active femaleness was used in >1-yr-old fish (Fig 1B). We surgically removed the testicular part of the digonic gonad in April (stage 6, post-spawning season). Histological data (H&E staining) showed that the surgery was successful and the fish only had an ovary. After the surgery, the primary oocytes gradually grew and then entered the vitellogenic oocyte stage in December (stage 9, pre-spawning season). Advanced oocyte diameters were 32.0 ± 1.21 μm in June (stage 6), 44.7 ± 0.08 μm in August (stage 7), 94.4 ± 8.94 μm in October (stage 7), and 132.0 ± 17.21 μm in December (stage 9). Similar to the ISH results (Fig 3A and 3B), qPCR data confirmed that the *gdf9* and *bmp15* transcripts were highly expressed in late primary oocytes (stage 7; October), but their expression was significantly lower in vitellogenic oocytes (stage 9; December) (Fig 3C). Taken together, these results showed that both *gdf9* and *bmp15* were specifically localized in oocytes and were expressed at high levels during primary oocyte growth.

### Oocyte-surrounding cells transformed from Sertoli cells to follicle-like cells in E2-induced ovotestes expression

**Fig 4:**
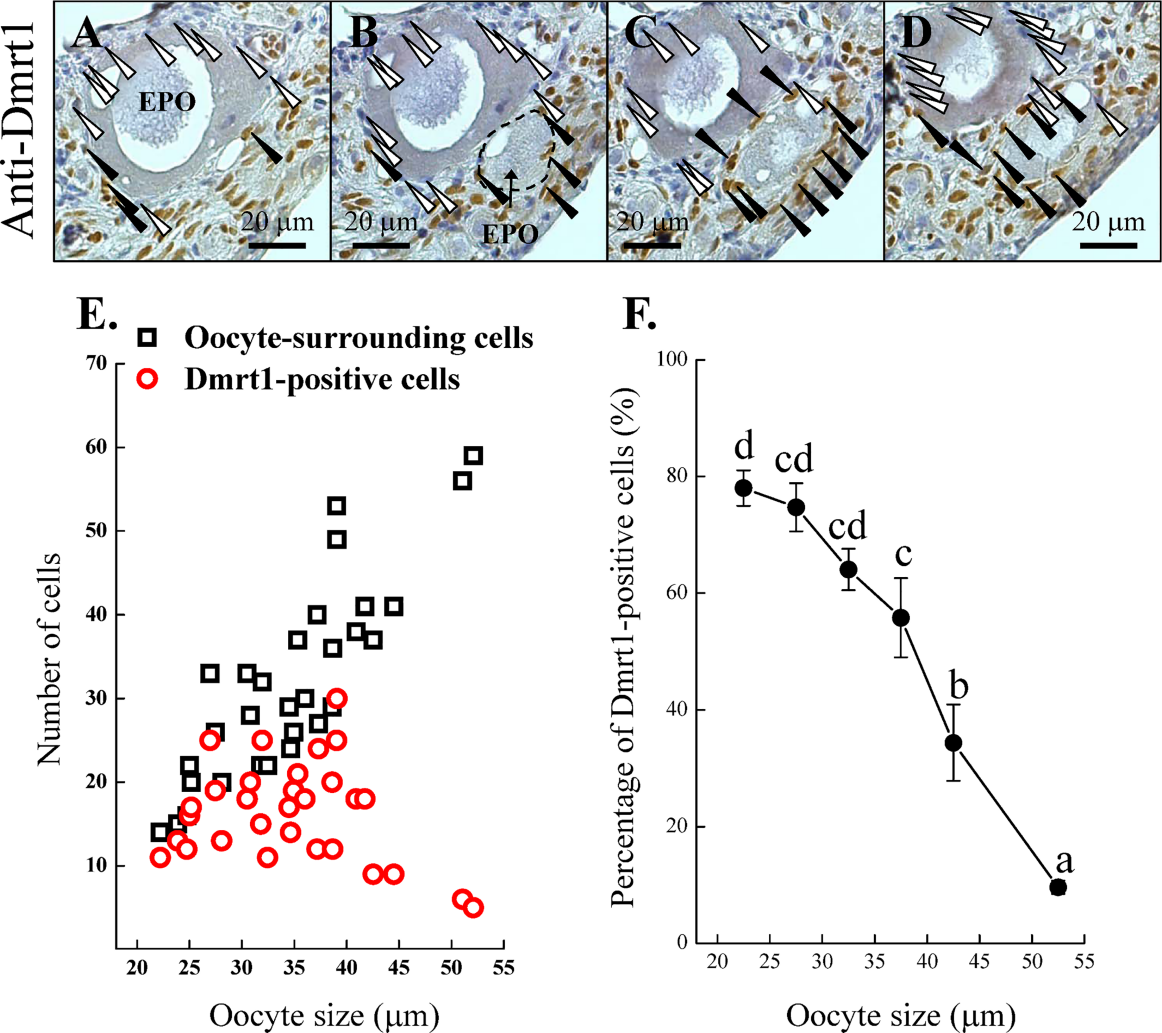
Ectopic oocyte-surrounding cell reprogramming during oocyte development. We created an ovotestis by ectopically inducing oocytes in the testicular region with estradiol (E2) administration and then E2 withdrawal. The Sertoli cells were confirmed by immunohistochemistry (IHC) for Dmrt1. Serial sections containing ectopic oocytes showed two different cell types surrounding the ectopic oocytes, Dmrt1-positive cells (Sertoli cells) and Dmrt1-negative cells (follicle-like cells) (A-D). Serial sections of various sizes of primary oocytes (30 ectopic oocytes) showed increasing numbers of oocyte-surrounding cells during oocyte growth (E). The percentage of Dmrt1-positive cells (Sertoli cells) was lower for large primary oocytes than for small primary oocytes (F). Black arrowheads indicate oocyte-surrounding Dmrt1-positive cells; white arrowheads indicate oocyte-surrounding Dmrt1-negative cells. Different small letters indicate significant differences (*P* < 0.05). EPO, ectopic primary oocyte.

We created an ovotestis by ectopically generating oocytes in the testicular region through exogenous E2 administration as described in our previous studies [9, 29] (Fig 1C). In previous studies, ectopic oocytes were surrounded by two types of somatic cells: Sertoli cells (Dmrt1 positive and Cyp19a1a negative) and follicle-like cells (Dmrt1 negative and Cyp19a1a positive) [9, 29]. To identify the cell types surrounding the ectopic oocytes in the ovotestes, we used a specific antibody for Dmrt1 (Sertoli cell marker) to identify the cell types (Dmrt1-positive for Sertoli cells and Dmrt1-negative for follicle-like cells) by IHC (Fig 4A–4D). IHC staining for Dmrt1 in serial sections revealed that small primary oocytes were surrounded by a large proportion of Dmrt1-positive cells (Sertoli cells) (Fig 4A–4D). In contrast, only a small proportion of Dmrt1-positive cells (considered Sertoli cells) were present around the large primary oocytes in the ovotestes (Fig 4A–4D), and the majority of the surrounding somatic cells (Dmrt1-negative) were considered follicle-like cells.

We further examined the reprogramming of oocyte-surrounding cells during oocyte growth. Our results from serial ovarian sections for various sizes of primary oocytes (30 ectopic oocytes in 5 ovotestes) revealed that the number of oocyte-surrounding cells increased during oocyte growth (Fig 4E). However, there were fewer Dmrt1-positive cells (Sertoli cells) around large primary oocytes than around small primary oocytes (Fig 4E). These results revealed that oocyte-surrounding cells were transformed from Sertoli cells to follicle-like cells during oocyte growth in the ovotestes (Fig 4F). Taken together, factors released by ectopic oocytes may stimulate transformation of the surrounding cells from Sertoli cells to follicle-like cells.

### Robust presence of *gdf9* and *bmp15* in ectopic oocytes in the ovotestes

**Fig 5:**
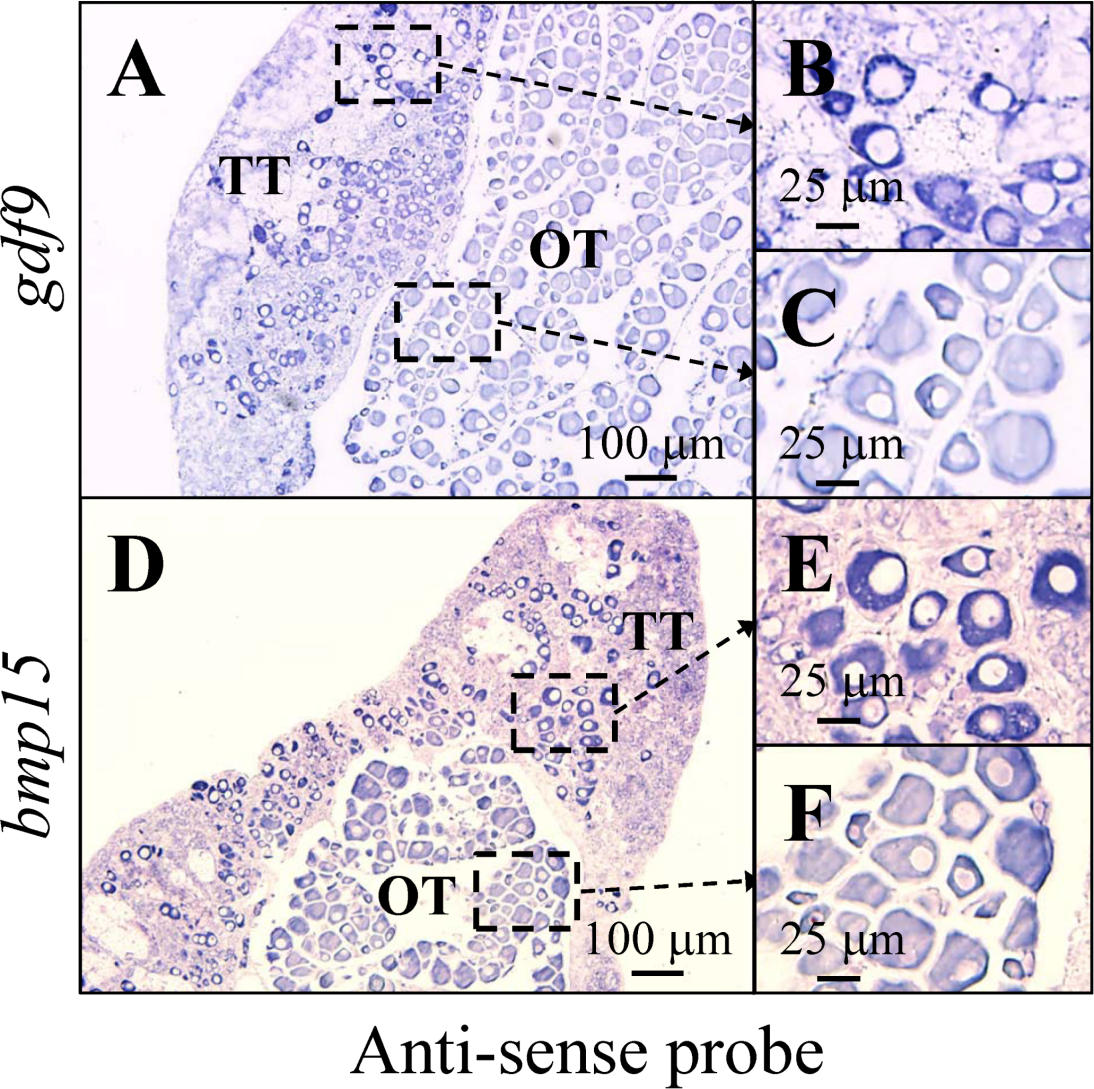
Differential expression of *gdf9* and *bmp15* in ectopic oocytes and normal oocytes. *gdf9* (A-C) and *bmp15* (D-F) mRNA expression was detected by *in situ* hybridization (ISH). Expression of *gdf9* was high in the ectopic oocytes of an ovotestis (A and B) compared with normal oocytes in the ovarian tissue (A and C). *bmp15* showed similar expression patterns to *gdf9*. Expression of *bmp15* was high in the ectopic oocytes of an ovotestis (D and E) compared with normal oocytes in the ovarian tissue (D and F). TT, testicular tissue, OT, ovarian tissue.

Both male and female germ cells were observed in the ovotestes (Fig 5A–5F). ISH staining showed that *gdf9* and *bmp15* were localized in the primary oocytes in the ovary and ovotestes (Fig 5A–5F). No signal was observed from sense probes for *gdf9* (S1I Fig) and *bmp15* (S1J Fig). Interestingly, ectopic oocytes in the ovotestes showed stronger expression of *gdf9* (Fig 5B) than the normal oocytes in the ovary (Fig 5C). Similar to the *gdf9* expression pattern, *bmp15* also showed more robust expression in ectopic oocytes in the ovotestes (Fig 5E) than in the normal oocytes in the ovary (Fig 5F). These data revealed that the differential expression of *gdf9* and *bmp15* between the ectopic oocytes (in the ovotestes) and normal oocytes (in the ovarian tissue) may play a role in reprogramming the cells surrounding the oocytes.

According to qPCR data, the relative expression order of oocyte-expressed genes (*figla*, *bmp15* and *gdf9*) was as follows: ovary (digonic gonad in status 7) > ovotestes > testes (digonic gonad in status 4) (S3 Fig). The presence of ectopic oocytes in the ovotestes caused the increased oocyte-expressed genes expression as compared to the testes. In contrast, the ovarian tissue of ectopic oocytes in the ovotestes was only a small portion in the ovotestes as compared to the testicular tissue; therefore, the ovary (the dignoic gonad in status 7) had a much higher oocyte-expressed genes expression than ovotestes (S3 Fig).

To further study the potential candidate genes in testis related to the presence of ectopic oocytes, we analyzed the gene expression patterns in testis of control fish and in regenerated testis (ovotestis; testis including ectopic oocytes) of E2-terminated fish. According to qPCR data, oocyte-expressed genes (*figla*, *gdf9*, and *bmp15*) had higher expression in the ovotestis of E2-terminated fish compared with in testis of control fish (S4 Fig). However, Sertoli cell markers (*dmrt1* and *amh*) and follicle cell markers (*foxl2* and *cyp19a1a*) had similar expression patterns in both groups (S4 Fig). Thus, our results revealed that Sertoli cell markers (*dmrt1* and *amh*) and follicle cell markers (*foxl2* and *cyp19a1a*) were not changed by the presence of ectopic oocytes in the ovotestis (testis-like) environment compared with the ovarian environment. The data further support the specific expression profiles of *figla*, *gdf9* and *bmp15* in the ectopic oocytes of ovotestes.

### Sex steroids did not stimulate *gdf9* and *bmp15* expression

**Fig 6:**
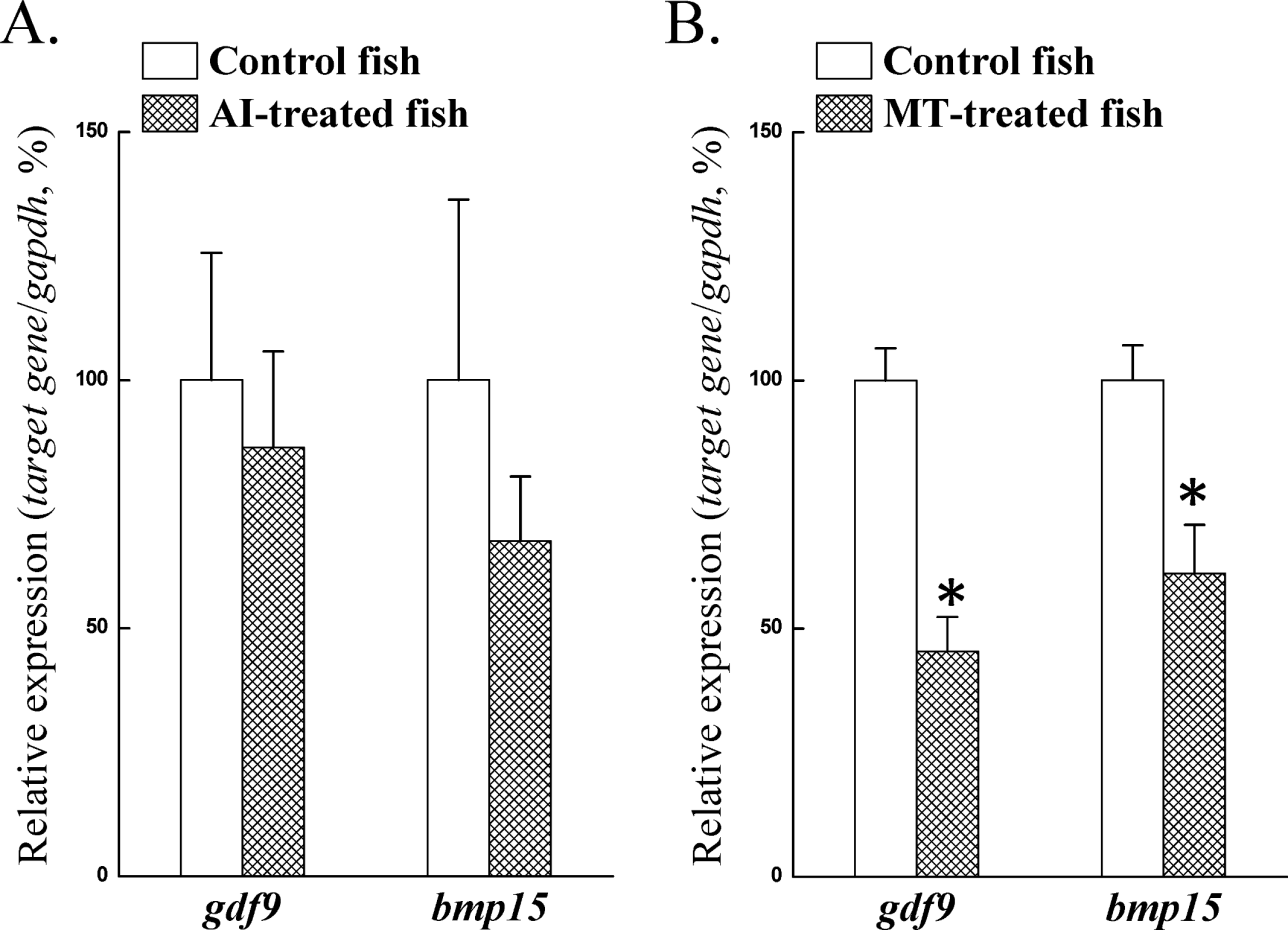
Relative gene expression of ovarian *gdf9* and *bmp15* after treatment with AI and MT. Fish were injected with AI (aromatase inhibitor, 5 mg/kg body weight) and MT (methyltestosterone, 1 mg/kg body weight) at day 0, 2, and 4 (n = 8). Gonads were collected 1 day after the third injection (day 5). The relative expression of *gdf9* and *bmp15* was analyzed by qPCR. Relative differences among control and treated groups were normalized to *gapdh*, and the highest value of the control group for each gene was defined as 100%. An asterisk indicates a significant difference between the control and treated groups (*P* < 0.05).

To further study the correlation between high *gdf9* and *bmp15* expression in ectopic oocytes and the testicular environment, we used AI (to reduce E2 levels) and MT (to elevate androgen levels) to determine the effects of sex steroids on gene expression. Fish at gonadal stage 7 were injected (i.p.) with AI (aromatase inhibitor, 1,4,6-androstatriene-3,17-dione; 5 mg/kg of body weight) or MT (methyltestosterone, 1 mg/kg of body weight). qPCR results revealed that AI treatment had no effect on *gdf9* and *bmp15* expression in the ovary (Fig 6A). Furthermore, MT treatment significantly decreased the *gdf9* and *bmp15* expression (Fig 6B). Taken together, our results revealed that *gdf9* and *bmp15* expression was not increased by inappropriate sex steroid levels (estrogens or androgens) in the ovotestis (testis-like) environment compared with the ovarian environment.

### Expression of *gdf9* and *bmp15* in oocytes was independent of Figla signaling

**Fig 7:**
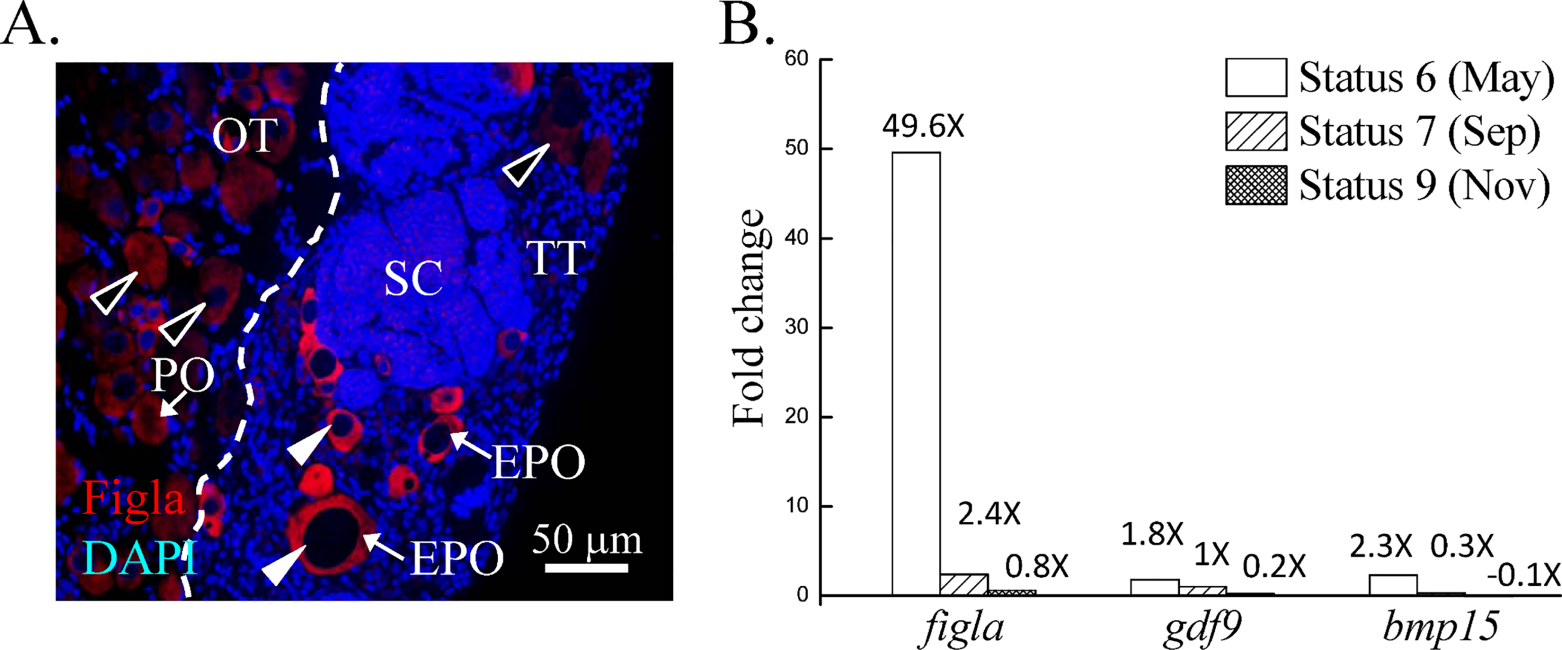
The relationship between Figla signaling and expression of *gdf9* and *bmp15*. We developed an *in vitro* culture system for oocytes to induce the Figla expression. The oocytes were transfected with the pcDNA3.1(+) vector (Invitrogen) for *figla* expression. Figla expression was prolonged in the ectopic oocytes in an ovotestis with the incorrect surrounding cells, as described in our previous work [28]. The prolonged Figla expression was confirmed by immunohistochemistry (IHC) (A). Neither *gdf9* nor *bmp15* expression showed a correlation with the fold change in *figla* expression in oocytes (B). The fold changes in *gdf9* and *bmp15* expression in the *figla*-induced group were calibrated based on the control group. Black arrowheads indicate the slight Figla expression in oocytes; white arrowheads indicate the robust Figla expression in oocytes. EPO, ectopic primary oocyte; PO, primary oocyte; SC, spermatocyte; OT, ovarian tissue; TT, testicular tissue.

Our previous study showed prolonged expression of *figla*/Figla in ectopic oocytes in the ovotestes compared with normal oocytes in the ovary [9]. High Figla expression was correlated with ectopic oocytes being surrounded with Sertoli cells [9]. In the present study, IF staining confirmed that there was a prolonged Figla expression in ectopic oocytes in the ovotestes compared with normal oocytes in the ovarian tissue (Fig 7A).

To investigate the correlation between *figla*/Figla expression and *gdf9* and *bmp15* expression in ectopic oocytes in the ovotestes, we developed an *in vitro* culture system for oocytes to induce *figla* expression. Oocytes were isolated based on a previously reported difference in adhesive ability between somatic cells and oocytes [32]. qPCR data showed higher *vasa* (germ cell marker) and *figla* (oocyte marker) expression and lower *foxl2* (follicle cell marker) expression in suspension cells than in the adhesive cells (data not shown). These data revealed that suspension cells are associated with oocytes. Successful vector delivery to oocytes was shown by PCR (S2A Fig). qPCR data revealed that vector-induced *figla* expression varied with gonadal stage (S2B Fig). Furthermore, qPCR data showed that the fold changes in *gdf9* and *bmp15* expression did not correlate with the changes in the *figla* expression levels (Fig 7B). Thus, the robust expression of *gdf9* and *bmp15* in ectopic oocytes in the ovotestes may have different roles than *figla* expression.

## Discussion

This study focused on how an ectopic oocyte in the ovotestes could create a female microenvironment through reprogramming of the surrounding cells from Sertoli cells to follicle-like cells. We suggest that oocytes may regulate somatic fate through Figla, Gdf9 and Bmp15 signaling.

### Ovarian *gdf9* and *bmp15* expression is associated with primary oocyte development in black porgy

Using qPCR, our study revealed that *gdf9* and *bmp15* were highly expressed in the ovary compared to the testes. ISH results further indicated that *gdf9* and *bmp15* were localized in oocytes. In addition, both *gdf9* and *bmp15* were most highly expressed at the primary oocyte stage, with expression gradually decreasing following the secondary oocyte growth stage. Similar to zebrafish [25], Gibel carp [23], ricefield eel (*Monopterus albus*) [33], and European sea bass [20, 21], *gdf9*/Gdf9 expression was high in oocytes at the primary oocyte stage and then gradually decreased during the secondary oocyte stage. In contrast, *bmp15*/Bmp15 expressions levels are variable among fish species. In zebrafish [24] and European seabass [20], *bmp15*/Bmp15 expression did not change during oocyte development. However, *bmp15* expression is high in primary oocytes stage in Gibel carp [22]. These data suggest that *gdf9*/Gdf9 has conserved functions at the primary oocyte stage, but *bmp15*/Bmp15 does not. Taken together, *gdf9* and *bmp15* were highly expressed in oocytes at the primary oocyte stage, and this may be related to a species-specific function in black porgy.

### Oocytes can differentiate under low plasma E2 levels

Previous work had shown that in black porgy, high endogenous E2 levels in plasma correlate with the sex change in the third reproductive cycle [4]. However, both ovary (primary oocyte stage) and testis can grow under the similarly low endogenous E2 levels in plasma [4, 10, 34]. Our present research and previous studies [7, 34] also showed that E2 administration did not maintain a stable female stage and that a reverse sex change (female-to-male) occurred after E2 withdrawal. Furthermore, AI (aromatase inhibitor) administration in 4-mo-old black porgy caused the regression of testicular tissue and further development of ovarian tissue; therefore, the blockage of Cyp19a1a activity by AI administration did not arrest the early ovarian development or early oocyte growth in juvenile black porgy [34]. In medaka, XX females lacking the function of a female germline-determining gene (*foxl3*) develop ovaries filled with functional sperm and oocytes [35]. In mammals, germ cells can undergo sustained differentiation outside of the gonad, and in ectopic sites, they all differentiate into oocytes, even in males [36]. Taken together, these findings suggest that oocytes could be developed in an E2-independent manner in some fish species.

### Oocytes are required for generating an appropriate female microenvironment

The present work and previous studies [9, 29] have shown that in black porgy, ectopic oocytes in ovotestes can reprogram the sexual fate of surrounding cells from Sertoli cells to follicle-like cells. These results revealed that ectopic oocytes could create an ovarian environment appropriate for follicle cell differentiation. The present study further showed that ectopic oocyte-surrounding cells were gradually transformed from Sertoli cells to follicle-like cells during oocyte growth. In addition, in protogynous wrasse [14], protogynous grouper [15] and tilapia (*Oreochromis niloticus*) [37], chemically induced oocyte depletion results in follicle cell alteration and sexual fate change from female to male soma during the female-to-male sex change. Similar data also showed that in zebrafish, adult females can sexually revert to fertile males after the loss of most of the germ cells [11]. These data suggest that the mechanism for reprogramming the cells surrounding oocytes is possibly determined by the oocytes themselves and their paracrine factors. The oocyte itself plays an important role in the development of the surrounding somatic cells.

### Both *gdf9* and *bmp15* may alter the sexual fate of oocyte-surrounding cells

Unlike most vertebrate clades, which have a stable sexual fate after the primary sex determination and gonadal differentiation, hermaphroditic fish have secondary sex determination (sex change) during their life [1, 28]. Secondary sex determination is precisely regulated by species-specific cues, including social factors (protogynous wrasse, protogynous anemone fish, and bi-directional sex change in gobiid fish), body size (protogynous grouper and protandrous ricefield eel), and age (protandrous sea bream) [1, 28]. Thus, a hermaphroditic fish requires constant maintenance of its primary sexual fate through sex-appropriate gene expression before sex change [28].

The present study showed that oocyte-surrounding cells (Sertoli cells) were gradually replaced by follicle-like cells during oocyte growth in the ovotestes. Moreover, in the present study, *gdf9* and *bmp15* expression levels were higher in the ectopic oocytes in the ovotestes than in normal oocytes in the ovary. In mammals, mutation in *Gdf9* results in decreased *Cyp19a1* expression [38]. Oocyte-derived *Gdf9* and *Bmp15* expression have been shown to be essential for ovarian follicle growth [16, 17]. Conversely, in zebrafish, all-male phenotypes are observed only with *bmp15* mutations and not with *gdf9* mutations [39]. These *bmp15* mutant females had normal ovary development but oocytes arrested at the primary oocyte stage, after which the oocytes degraded, and the sexual fate switched to maleness [39]. Furthermore, these primary oocyte-surrounded granulosa cells did not express *cyp19a1a* [39]. Thus, *bmp15* is suggested to have dual functions in zebrafish: maintenance of the female fate and the prevention of oocyte maturation [26, 27]. Taken together, these differences in function between mammals and fishes may be due to fish having two paralogs of aromatase: *cyp19a1a* in gonad and *cyp19a1b* in brain [3, 5]. Therefore, the robust oocyte-derived *gdf9* and *bmp15* expression may play important roles in altering the fate of oocyte-surrounding cells from male to female soma in the ovotestes in black porgy.

### The expression of *gdf9* and *bmp15* in oocytes, not the levels of sex steroids, is related to the types of surrounding cells

Our previous studies showed that oocyte-specific *figla*/Figla expression is associated with the types of surrounding cells [9]. Prolonged Figla expression was found in ectopic oocytes surrounded by Sertoli cells but not follicle-like cells in the ovotestes [9]. Furthermore, sex steroid treatment did not influence *figla* expression in the ovary [9]. The present study showed patterns of *gdf9* and *bmp15* expression similar that of *figla* expression. Robust *gdf9* and *bmp15* expression was observed in ectopic oocytes surrounded by Sertoli cells in the ovotestes. Neither *gdf9* expression nor *bmp15* expression was induced by AI or MT treatment. Therefore, this robust *gdf9* and *bmp15* expression in ectopic oocytes may be induced by oocytes surrounded by Sertoli cells but not in the inappropriate microenvironment (i.e., sex steroid levels) of the testis. We suggest that this unique expression pattern of *gdf9 and bmp15* in ectopic oocytes is regulated by the types of surrounding cells.

### Ectopic oocyte-surrounding cell reprogramming occurs by both Figla-dependent and Figla-independent means

**Fig 8:**
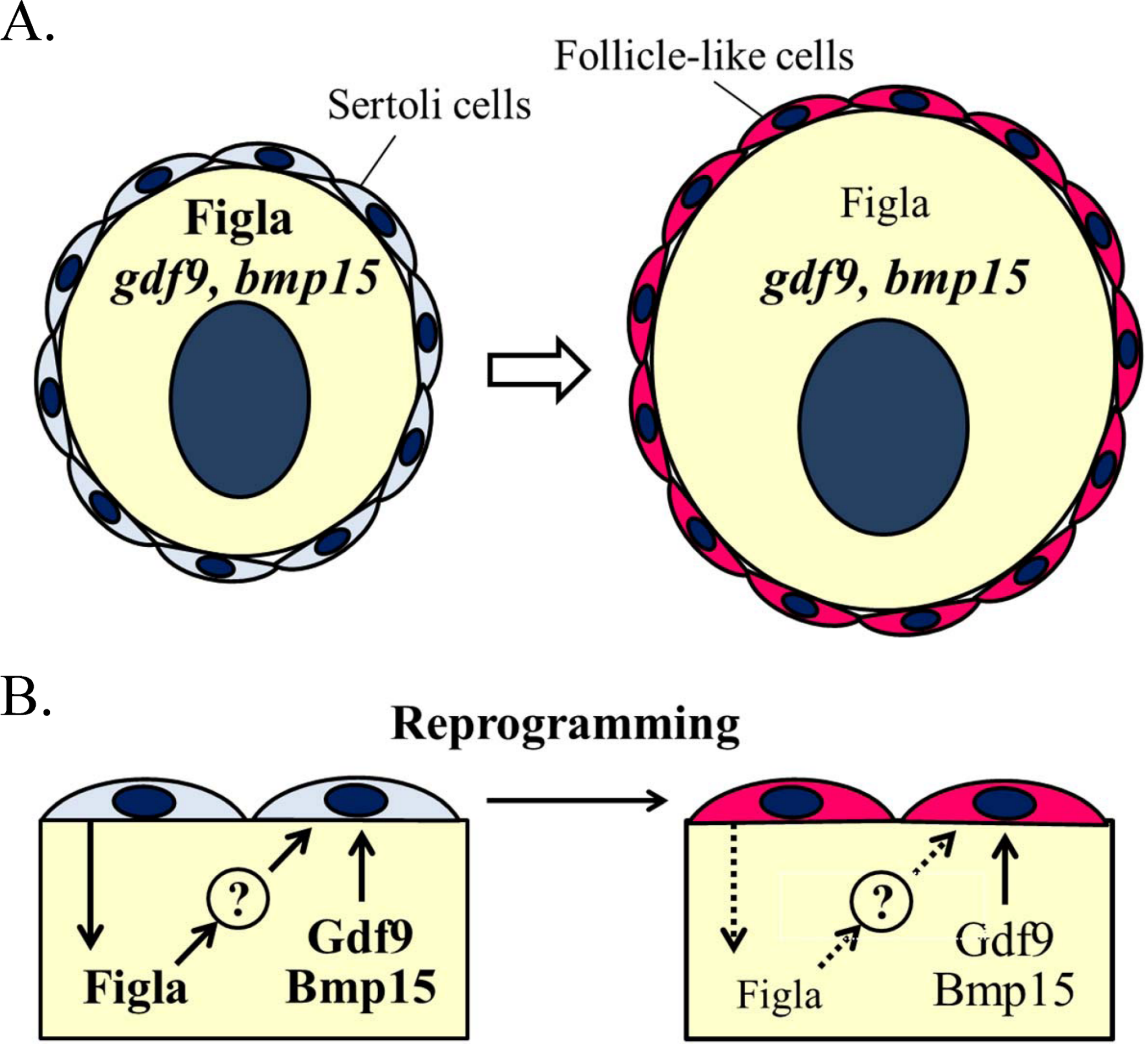
Model for reprogramming the cells surrounding ectopic oocytes. Ectopic oocytes can alter the fate of somatic cells from Sertoli cells to follicle-like cells. Both prolonged Figla expression and robust *gdf9* and *bmp15* expression were associated with Sertoli cell-surrounded ectopic oocytes (A). High *gdf9* and *bmp15* and low *Figla* expression are observed in the primary oocytes surrounding follicle cells (A). We demonstrated that robust expression of *gdf9* and *bmp15* was associated with ectopic oocytes surrounded by Sertoli cells but not by the inappropriately male microenvironment. Our data suggest that there are two independent pathways for the conversion of oocyte-surrounding cells from male somatic fate to female somatic fate: Figla-dependent and Figla-independent (B).

Our previous study showed that *figla*/Figla expression was associated with ovarian differentiation and the reprogramming of ectopic oocyte-surrounding cells [9, 10]. In mice, *Figla* is a key factor in coordinating the expression of ovary-specific genes [40, 41]. *Figla* also represses the expression of male-associated genes during oogenesis in mice [40]. As in mammals, *figla* is important for ovarian differentiation in fish (zebrafish: [42]; medaka: [43]; tilapia: [44]). Thus, *figla* may play an important role in ovarian differentiation and follicle formation in black porgy. However, Figla is an oocyte-expressed transcription factor. Thus, this Figla-dependent mechanism needs oocyte-releasing paracrine signaling to alter the soma fate from male to female. The present study demonstrated that *gdf9* and *bmp15* were robustly expressed in ectopic oocytes (Fig 5 and Fig 8A). We also found that the robust *figla*, *gdf9* and *bmp15* expressions but not other examined sex-related genes (*dmrt1*, *foxl2*, *cyp19a1a*) were all specifically detected in the ovotestes (the testes containing ectopic oocytes surrounding Sertoli cells). However, the *in vitro* data in this study showed that the expression of *gdf9* and *bmp15* was not induced by high *figla* expression. In zebrafish, *gdf9*-deficient female had normally ovary [39]. In contrast, *bmp15*-deficient female alter sex from female to male during the juvenile stage [39]. Furthermore, Sertoli cell-expressed *amh* were significantly suppressed by recombinant Gdf9 in zebrafish [45]. Taken together, both *gdf9* and *bmp15* are suggested to involve in different mechanisms (the stimulation of the follicle cell characteristics and/or the suppression of the Sertoli cell characteristics) in black porgy.

Our data seems to support the positive relationship in the expression of *gdf9* and *bmp15* (Figs 2, 3, 5 and 6; S3 Fig). More studies with special approaches are needed to further understand the specific roles and expression of *gdf9* and *bmp15* in the reprogramming sexual fate of oocytes surrounding cells during oocyte development in the testes. Thus, our data suggest that the process of reprogramming the surrounding cells includes at least two parallel pathways: a Figla-dependent mechanism and a Figla-independent mechanism (Fig 8B).

## Conclusions

We demonstrated that *gdf9* and *bmp15* were localized in primary oocytes and that their expression gradually decreased after oocytes entered the secondary oocyte stage. The data indicated that robust expression of *gdf9* and *bmp15* was associated with ectopic oocytes surrounded by Sertoli cells but not with the inappropriately male microenvironment. Our data also indicated that there are two independent pathways for the conversion of oocyte-surrounding cells from the male somatic fate to the female somatic fate. This functional switch might clarify how fish alter their sexual fate from one sex to the other during the transition from gonochorism to hermaphroditism.

## Supporting information

S1 Checklist (PDF)

S1 Fig References for *in situ* hybridization (ISH) in the different gonad stages. The references for ISH were confirmed by using sense probes for *gdf9* and *bmp15*. No signal was observed with the sense probes for *gdf9* (A-C) and *bmp15* (D-F) at stage 7, stage 4, and stage 5 for Fig 2. No signal was observed with the sense probes for *gdf9* (G) and *bmp15* (H) at stage 9 for Fig 3. No signal was observed with the sense probes for *gdf9* (I) and *bmp15* (J) in testis for Fig 5. CT, connective tissue; OT, ovarian tissue; RTT, regressed testicular tissue; TT, testicular tissue.
(TIFF)

S2 Fig *In vitro figla*-induced expression in oocyte culture system. We developed an *in vitro* oocyte culture system to induce *figla* expression by expression vectors. Successful vector delivery was confirmed by PCR (A). no. 1 = DNA isolation from vector delivered oocytes, no. 2 = DNA isolation from oocytes without vector delivery, no. 3 = negative control, no. 4 = vector. Differential *figla* expression was shown among different gonadal stages (stage 6, stage 7, and stage 9) after the vector delivery (B).
(TIFF)

S3 Fig The relative oocyte-expressed genes expression in the testis, ovary and E2-induced ovotestis. We created an ovotestis by ectopically inducing oocytes in the testicular region with estradiol (E2) administration and then E2 withdrawal. Testis (the digonic gonad in status 4, n = 8), E2-induced ovotestis (testis with ectopic oocytes, n = 4) and ovary (the digonic gonad in status 7, n = 6) were used for qPCR analysis. Oocyte-expressed genes (*figla*, *bmp15* and *gdf9*) were analyxed by PCR. The gene value in the ovary was defined as 100%. Different small letters indicate significant difference (*P* <0.05).
(TIFF)

S4 Fig The relative genes expression in the testis and E2-induced ovotestis. We created an ovotestis by ectopically inducing oocytes in the testicular region with estradiol (E2) administration and then E2 withdrawal. Normal testis (n = 8) and E2-induced ovotestis (n = 4) were used for RNA analysis. qPCR data confirmed that oocytes-expressed *figla*, *gdf9*, and *bmp15* were expressed at higher levels in the ovotestes than in the testes. No difference of Sertoli cells marker (*dmrt1* and *amh*) and follicle cells marker (*foxl2* and *cyp19a1a*) were observed in the ovotestes than in the testes. An asterisk indicates a significant difference between the testes and ovotestis (P < 0.05).
(TIFF)

## References

[1] Avise JC, Mank JE. Evolutionary perspectives on hermaphroditism in fishes. Sex Dev. 2009; 3:152–163. doi: 10.1159/000223079 19684459 10.1159/000223079

[2] Devlin RH, Nagahama Y. Sex determination and sex differentiation in fish: an overview of genetic, physiological, and environmental influences. Aquaculture. 2002; 208:191–364.

[3] Guiguen Y, Fostier A, Piferrer F, Chang CF. Ovarian aromatase and estrogens: a pivotal role for gonadal sex differentiation and sex change in fish. Gen Comp Endocrinol. 2010; 165:352–366. doi: 10.1016/j.ygcen.2009.03.002 19289125 10.1016/j.ygcen.2009.03.002

[4] Chang CF, Lee MF, Chen GR. Estradiol-17β associated with the sex reversal in protandrous black porgy, *Acanthopagrus schlegeli*. J Exp Zool. 1994; 268:53–58.

[5] Diotel N, Le Page Y, Mouriec K, Tong SK, Pellegrini E, Vaillant C, et al Aromatase in the brain of teleost fish: expression, regulation and putative functions. Front Neuroendocrinol. 2010; 31:172–192. doi: 10.1016/j.yfrne.2010.01.003 20116395 10.1016/j.yfrne.2010.01.003

[6] Hecker M, Murphy MB, Coady KK, Villeneuve DL, Jones PD, Carr JA, Solomon KR, Smith EE, Van Der Kraak G, Gross T, Du Preez L, Kendall RJ, et al Terminology of gonadal anomalies in fish and amphibians resulting from chemical exposures. Rev Environ Contam Toxicol. 2006; 187:103–131. 16802580

[7] Lee YH, Yueh WS, Du JL, Sun LT, Chang CF. Aromatase inhibitors block natural sex change and induce male function in the protandrous black porgy, *Acanthopagrus schlegeli* Bleeker: possible mechanism of natural sex change. Biol Reprod. 2002; 66:1749–1754. 12021057 10.1095/biolreprod66.6.1749

[8] Nishimura T, Herpin A, Kimura T, Hara I, Kawasaki T, Nakamura S, et al Analysis of a novel gene, Sdgc, reveals sex chromosome-dependent differences of medaka germ cells prior to gonad formation. Development. 2014; 141:3363–3369. doi: 10.1242/dev.106864 25078651 10.1242/dev.106864

[9] Wu GC, Chang CF. Oocytes survive in the testis by altering the soma fate from male to female in the protandrous black porgy, *Acanthopagrus schlegeli*. Biol Reprod. 2013; 88:19 doi: 10.1095/biolreprod.112.104398 23197163 10.1095/biolreprod.112.104398

[10] Wu GC, Tomy S, Nakamura M, Chang CF. Dual roles of cyp19a1a in gonadal sex differentiation and development in the protandrous black porgy, *Acanthopagrus schlegeli*. Biol Reprod. 2008; 79:1111–1120. doi: 10.1095/biolreprod.108.069146 18667752 10.1095/biolreprod.108.069146

[11] Dranow DB, Tucker RP, Draper BW. Germ cells are required to maintain a stable sexual phenotype in adult zebrafish. Dev Biol. 2013; 376:43–50. doi: 10.1016/j.ydbio.2013.01.016 23348677 10.1016/j.ydbio.2013.01.016

[12] Siegfried KR, Nusslein-Volhard C. Germ line control of female sex determination in zebrafish. Dev Biol. 2008; 324:277–287. doi: 10.1016/j.ydbio.2008.09.025 18930041 10.1016/j.ydbio.2008.09.025

[13] Kurokawa H, Saito D, Nakamura S, Katoh-Fukui Y, Ohta K, Baba T, et al Germ cells are essential for sexual dimorphism in the medaka gonad. Proc Natl Acad Sci U S A. 2007; 104:16958–16963. doi: 10.1073/pnas.0609932104 17940041 10.1073/pnas.0609932104 PMC2040408

[14] Nozu R, Horiguchi R, Murata R, Kobayashi Y, Nakamura M. Survival of ovarian somatic cells during sex change in the protogynous wrasse, *Halichoeres trimaculatus*. Fish Physiol Biochem. 2013; 39:47–51. doi: 10.1007/s10695-012-9632-2 22422286 10.1007/s10695-012-9632-2

[15] Wu GC, Tey WG, Li HW, Chang CF. Sexual Fate Reprogramming in the Steroid-Induced Bi-Directional Sex Change in the Protogynous Orange-Spotted Grouper, *Epinephelus coioides*. PLoS One. 2015; 10:e0145438 doi: 10.1371/journal.pone.0145438 26714271 10.1371/journal.pone.0145438 PMC4694621

[16] Juengel JL, Bodensteiner KJ, Heath DA, Hudson NL, Moeller CL, Smith P, et al Physiology of GDF9 and BMP15 signalling molecules. Anim Reprod Sci. 2004; 82–83:447–460. doi: 10.1016/j.anireprosci.2004.04.021 15271472 10.1016/j.anireprosci.2004.04.021

[17] Paulini F, Melo EO. The role of oocyte-secreted factors GDF9 and BMP15 in follicular development and oogenesis. Reprod Domest Anim. 2011; 46:354–361. doi: 10.1111/j.1439-0531.2010.01739.x 21198974 10.1111/j.1439-0531.2010.01739.x

[18] Mottershead DG, Ritter LJ, Gilchrist RB. Signalling pathways mediating specific synergistic interactions between GDF9 and BMP15. Mol Hum Reprod. 2012; 18:121–128. doi: 10.1093/molehr/gar056 21911477 10.1093/molehr/gar056 PMC3292392

[19] Zhang Y, Yuan C, Qin F, Hu G, Wang Z. Molecular characterization of gdf9 and bmp15 genes in rare minnow *Gobiocypris rarus* and their expression upon bisphenol A exposure in adult females. Gene. 2014; 546:214–221. doi: 10.1016/j.gene.2014.06.013 24914497 10.1016/j.gene.2014.06.013

[20] Garcia-Lopez A, Sanchez-Amaya MI, Halm S, Astola A, Prat F. Bone morphogenetic protein 15 and growth differentiation factor 9 expression in the ovary of European sea bass (*Dicentrarchus labrax*): cellular localization, developmental profiles, and response to unilateral ovariectomy. Gen Comp Endocrinol. 2011; 174:326–334. doi: 10.1016/j.ygcen.2011.09.011 21978589 10.1016/j.ygcen.2011.09.011

[21] Halm S, Ibanez AJ, Tyler CR, Prat F. Molecular characterisation of growth differentiation factor 9 (gdf9) and bone morphogenetic protein 15 (bmp15) and their patterns of gene expression during the ovarian reproductive cycle in the European sea bass. Mol Cell Endocrinol. 2008; 291:95–103. doi: 10.1016/j.mce.2008.03.002 18423979 10.1016/j.mce.2008.03.002

[22] Chen AQ, Liu ZW, Yang ZG, Leng XJ. Characterization of bmp15 and its regulation by human chorionic gonadotropin in the follicle of gibel carp (*Carassius auratus gibelio*). Comp Biochem Physiol B Biochem Mol Biol. 2012; 163:121–128. doi: 10.1016/j.cbpb.2012.05.009 22613815 10.1016/j.cbpb.2012.05.009

[23] Liu Z, Chen A, Yang Z, Wei H, Leng X. Molecular characterization of growth differentiation factor 9 and its spatio-temporal expression pattern in gibel carp (*Carassius auratus gibelio*). Mol Biol Rep. 2012; 39:3863–3870. doi: 10.1007/s11033-011-1165-8 21779806 10.1007/s11033-011-1165-8

[24] Clelland E, Kohli G, Campbell RK, Sharma S, Shimasaki S, Peng C. Bone morphogenetic protein-15 in the zebrafish ovary: complementary deoxyribonucleic acid cloning, genomic organization, tissue distribution, and role in oocyte maturation. Endocrinology. 2006; 147:201–209. doi: 10.1210/en.2005-1017 16210364 10.1210/en.2005-1017

[25] Liu L, Ge W. Growth differentiation factor 9 and its spatiotemporal expression and regulation in the zebrafish ovary. Biol Reprod. 2007; 76:294–302. doi: 10.1095/biolreprod.106.054668 17093199 10.1095/biolreprod.106.054668

[26] Clelland ES, Tan Q, Balofsky A, Lacivita R, Peng C. Inhibition of premature oocyte maturation: a role for bone morphogenetic protein 15 in zebrafish ovarian follicles. Endocrinology. 2007; 148:5451–5458. doi: 10.1210/en.2007-0674 17656459 10.1210/en.2007-0674

[27] Peng C, Clelland E, Tan Q. Potential role of bone morphogenetic protein-15 in zebrafish follicle development and oocyte maturation. Comp Biochem Physiol A Mol Integr Physiol. 2009; 153:83–87. doi: 10.1016/j.cbpa.2008.09.034 18951993 10.1016/j.cbpa.2008.09.034

[28] Wu GC, Chang CF. The switch of secondary sex determination in protandrous black porgy, *Acanthopagrus schlegeli*. Fish Physiol Biochem. 2013; 39:33–38. doi: 10.1007/s10695-012-9618-0 22411079 10.1007/s10695-012-9618-0

[29] Wu GC, Li HW, Luo JW, Chen C, Chang CF. The Potential Role of Amh to Prevent Ectopic Female Development in Testicular Tissue of the Protandrous Black Porgy, *Acanthopagrus schlegelii*. Biol Reprod. 2015; 92:158 doi: 10.1095/biolreprod.114.126953 25855263 10.1095/biolreprod.114.126953

[30] Wu GC, Chang CF. wnt4 Is associated with the development of ovarian tissue in the protandrous black Porgy, *Acanthopagrus schlegeli*. Biol Reprod. 2009; 81:1073–1082. doi: 10.1095/biolreprod.109.077362 19605791 10.1095/biolreprod.109.077362

[31] Wu GC, Chiu PC, Lin CJ, Lyu YS, Lan DS, Chang CF. Testicular dmrt1 is involved in the sexual fate of the ovotestis in the protandrous black porgy. Biol Reprod. 2012; 86:41 doi: 10.1095/biolreprod.111.095695 22034528 10.1095/biolreprod.111.095695

[32] Wu GC, Li HW, Huang CH, Lin HJ, Lin CJ, Chang CF. The Testis Is a Primary Factor That Contributes to Epigenetic Modifications in the Ovaries of the Protandrous Black Porgy, *Acanthopagrus schlegelii*. Biol Reprod. 2016; 94:132 doi: 10.1095/biolreprod.115.137463 27103447 10.1095/biolreprod.115.137463

[33] He Z, Wu Y, Xie J, Wang T, Zhang L, Zhang W. Growth differentiation factor 9 (Gdf9) was localized in the female as well as male germ cells in a protogynous hermaphroditic teleost fish, ricefield eel *Monopterus albus*. Gen Comp Endocrinol. 2012; 178:355–362. doi: 10.1016/j.ygcen.2012.06.016 22732078 10.1016/j.ygcen.2012.06.016

[34] Wu GC, Tomy S, Chang CF. The expression of nr0b1 and nr5a4 during gonad development and sex change in protandrous black porgy fish, *Acanthopagrus schlegeli*. Biol Reprod. 2008; 78:200–210. doi: 10.1095/biolreprod.107.062612 17959853 10.1095/biolreprod.107.062612

[35] Nishimura T, Sato T, Yamamoto Y, Watakabe I, Ohkawa Y, Suyama M, et al Sex determination. foxl3 is a germ cell-intrinsic factor involved in sperm-egg fate decision in medaka. Science. 2015; 349:328–331. doi: 10.1126/science.aaa2657 26067255 10.1126/science.aaa2657

[36] Upadhyay S, Zamboni L. Ectopic germ cells: natural model for the study of germ cell sexual differentiation. Proc Natl Acad Sci U S A. 1982; 79:6584–6588. 6959138 10.1073/pnas.79.21.6584 PMC347172

[37] Sun LN, Jiang XL, Xie QP, Yuan J, Huang BF, Tao WJ, et al Transdifferentiation of differentiated ovary into functional testis by long-term treatment of aromatase inhibitor in Nile tilapia. Endocrinology. 2014; 155:1476–1488. doi: 10.1210/en.2013-1959 24437491 10.1210/en.2013-1959

[38] Dong J, Albertini DF, Nishimori K, Kumar TR, Lu N, Matzuk MM. Growth differentiation factor-9 is required during early ovarian folliculogenesis. Nature. 1996; 383:531–535. doi: 10.1038/383531a0 8849725 10.1038/383531a0

[39] Dranow DB, Hu K, Bird AM, Lawry ST, Adams MT, Sanchez A, et al Bmp15 Is an Oocyte-Produced Signal Required for Maintenance of the Adult Female Sexual Phenotype in Zebrafish. PLoS Genet. 2016; 12:e1006323. 10.1371/journal.pgen.1006323 PMC5028036 27642754

[40] Hu W, Gauthier L, Baibakov B, Jimenez-Movilla M, Dean J. FIGLA, a basic helix-loop-helix transcription factor, balances sexually dimorphic gene expression in postnatal oocytes. Mol Cell Biol. 2010; 30:3661–3671. doi: 10.1128/MCB.00201-10 20479125 10.1128/MCB.00201-10 PMC2897557

[41] Joshi S, Davies H, Sims LP, Levy SE, Dean J. Ovarian gene expression in the absence of FIGLA, an oocyte-specific transcription factor. BMC Dev Biol. 2007; 7:67 doi: 10.1186/1471-213X-7-67 17567914 10.1186/1471-213X-7-67 PMC1906760

[42] Jørgensen A, Morthorst JE, Andersen O, Rasmussen LJ, Bjerregaard P. Expression profiles for six zebrafish genes during gonadal sex differentiation. Reprod Biol Endocrinol. 2008; 6:25 doi: 10.1186/1477-7827-6-25 18590525 10.1186/1477-7827-6-25 PMC2500022

[43] Kanamori A, Toyama K, Kitagawa S, Kamehara A, Higuchi T, Kamachi Y, et al Comparative genomics approach to the expression of figα, one of the earliest marker genes of oocyte differentiation in medaka (*Oryzias latipes*). Gene. 2008; 423:180–187. doi: 10.1016/j.gene.2008.07.007 18678233 10.1016/j.gene.2008.07.007

[44] Qiu Y, Sun S, Charkraborty T, Wu L, Sun L, Wei J, et al Figla Favors Ovarian Differentiation by Antagonizing Spermatogenesis in a Teleosts, Nile Tilapia (*Oreochromis niloticus*). PLoS One. 2015; 10:e0123900 doi: 10.1371/journal.pone.0123900 25894586 10.1371/journal.pone.0123900 PMC4404364

[45] Chen W, Liu L, Ge W. Expression analysis of growth differentiation factor 9 (Gdf9/gdf9), anti-mullerian hormone (Amh/amh) and aromatase (Cyp19a1a/cyp19a1a) during gonadal differentiation of the zebrafish, Danio rerio. Biol Reprod. 2017; 96:401–413. doi: 10.1095/biolreprod.116.144964 28203731 10.1095/biolreprod.116.144964

