# Supplementary figures and images for: Robust gdf9 and bmp15 expression in the oocytes of ovotestes through the Figla-independent pathway in the hermaphroditic black porgy, Acanthopagrus schlegelii
Source: PLoS One. 2017 Oct 26;12(10):e0186991. doi: 10.1371/journal.pone.0186991 (PMC5658113; doi:10.1371/journal.pone.0186991)

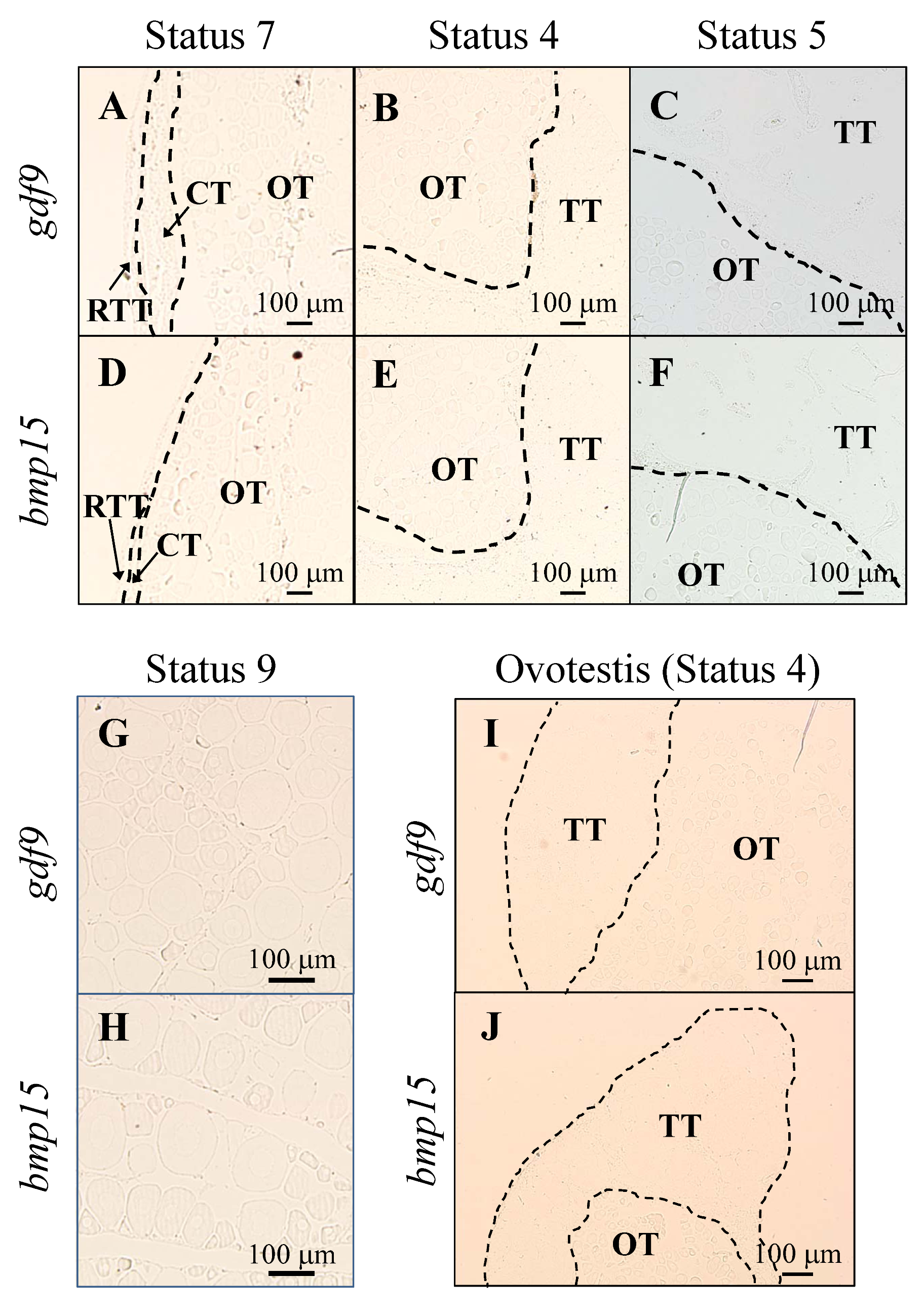

Supplement: S1 Fig — The references for ISH were confirmed by using sense probes for gdf9 and bmp15. No signal was observed with the sense probes for gdf9 (A-C) and bmp15 (D-F) at stage 7, stage 4, and stage 5 for Fig 2. No signal was observed with the sense probes for gdf9 (G) and bmp15 (H) at stage 9 for Fig 3. No signal was observed with the sense probes for gdf9 (I) and bmp15 (J) in testis for Fig 5. CT, connective tissue; OT, ovarian tissue; RTT, regressed testicular tissue; TT, testicular tissue. (TIFF) [file pone.0186991.s002.tiff]

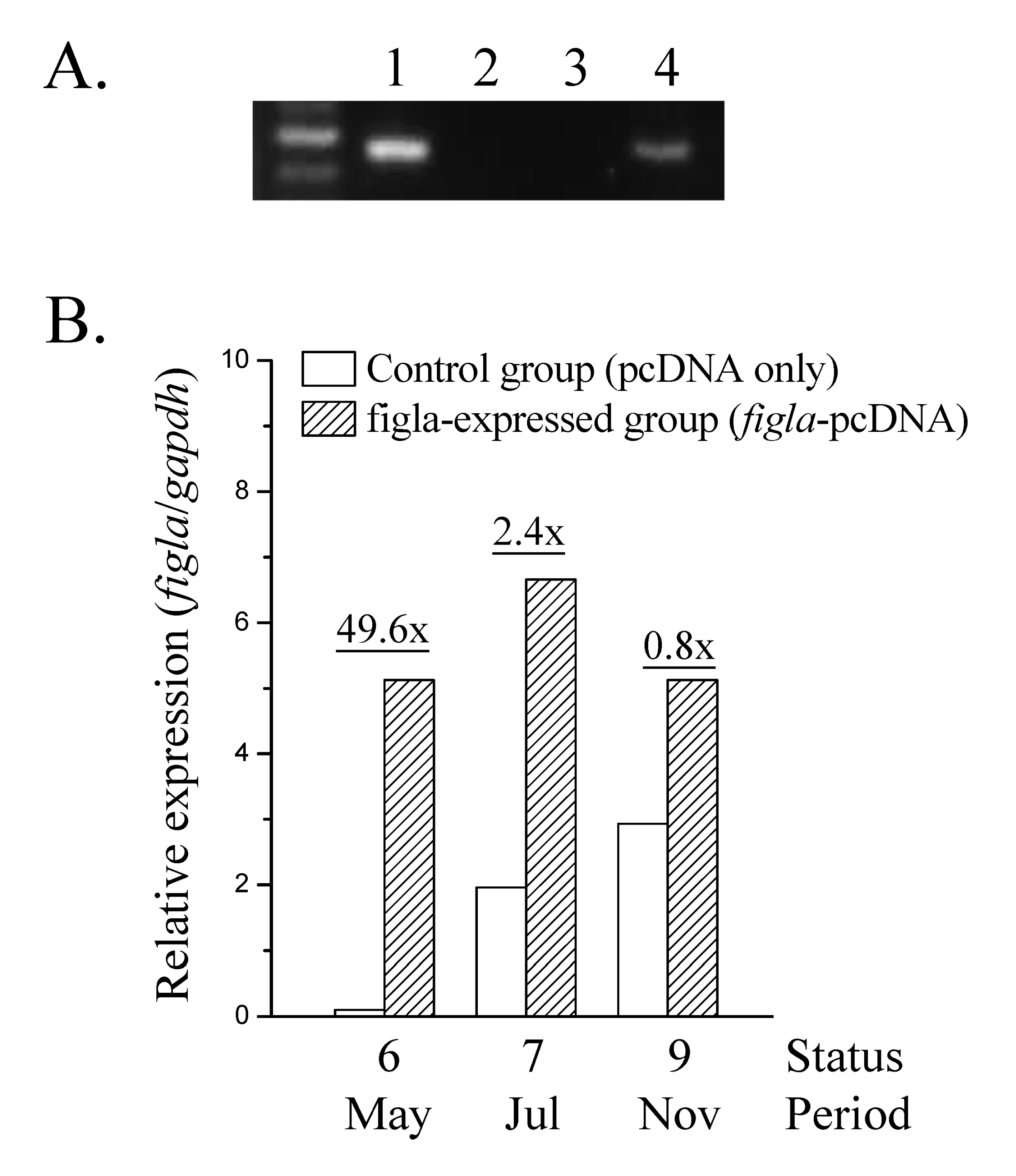

Supplement: S2 Fig — We developed an in vitro oocyte culture system to induce figla expression by expression vectors. Successful vector delivery was confirmed by PCR (A). no. 1 = DNA isolation from vector delivered oocytes, no. 2 = DNA isolation from oocytes without vector delivery, no. 3 = negative control, no. 4 = vector. Differential figla expression was shown among different gonadal stages (stage 6, stage 7, and stage 9) after the vector delivery (B). (TIFF) [file pone.0186991.s003.tiff]

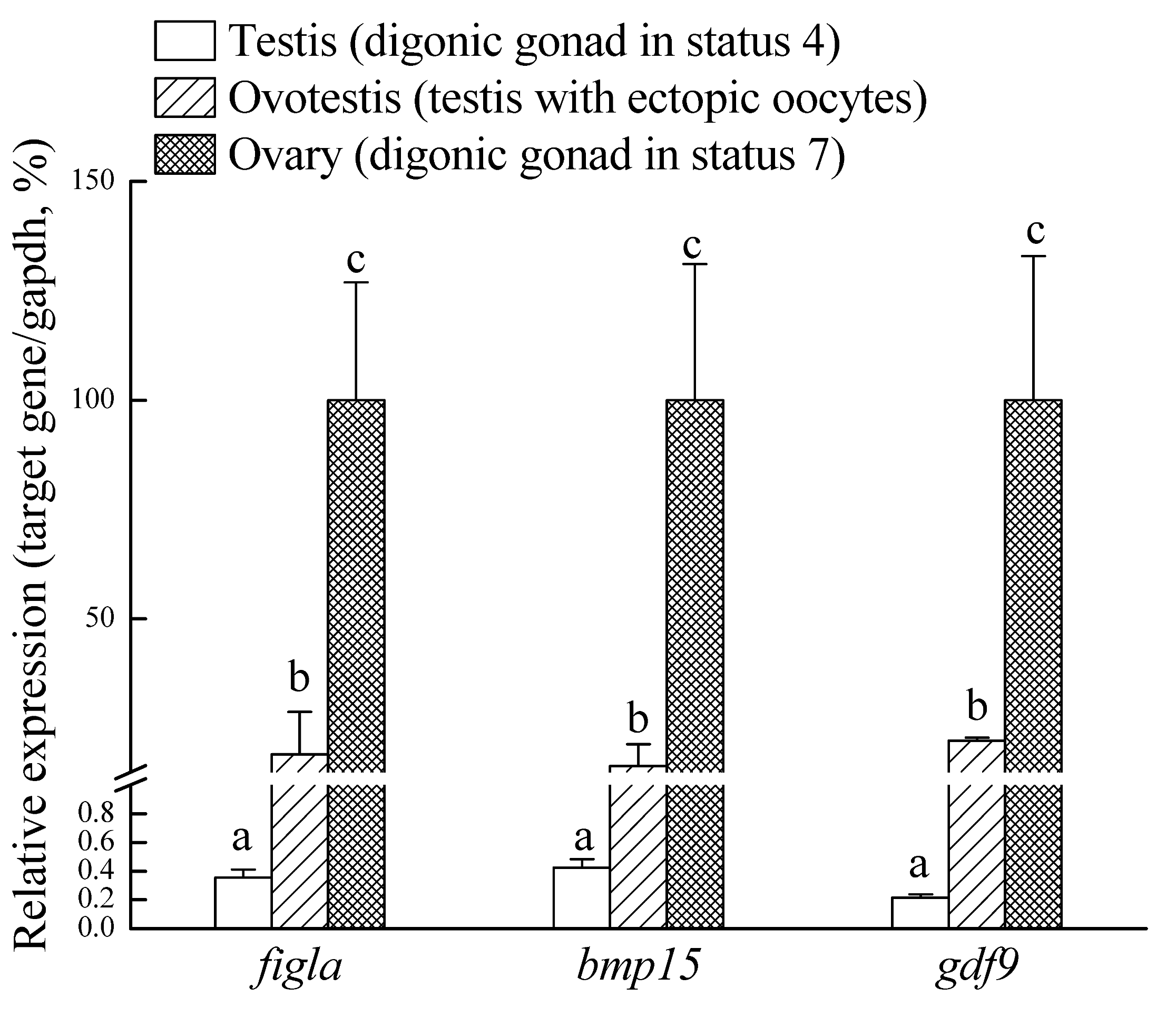

Supplement: S3 Fig — We created an ovotestis by ectopically inducing oocytes in the testicular region with estradiol (E2) administration and then E2 withdrawal. Testis (the digonic gonad in status 4, n = 8), E2-induced ovotestis (testis with ectopic oocytes, n = 4) and ovary (the digonic gonad in status 7, n = 6) were used for qPCR analysis. Oocyte-expressed genes (figla, bmp15 and gdf9) were analyxed by PCR. The gene value in the ovary was defined as 100%. Different small letters indicate significant difference (P <0.05). (TIFF) [file pone.0186991.s004.tiff]

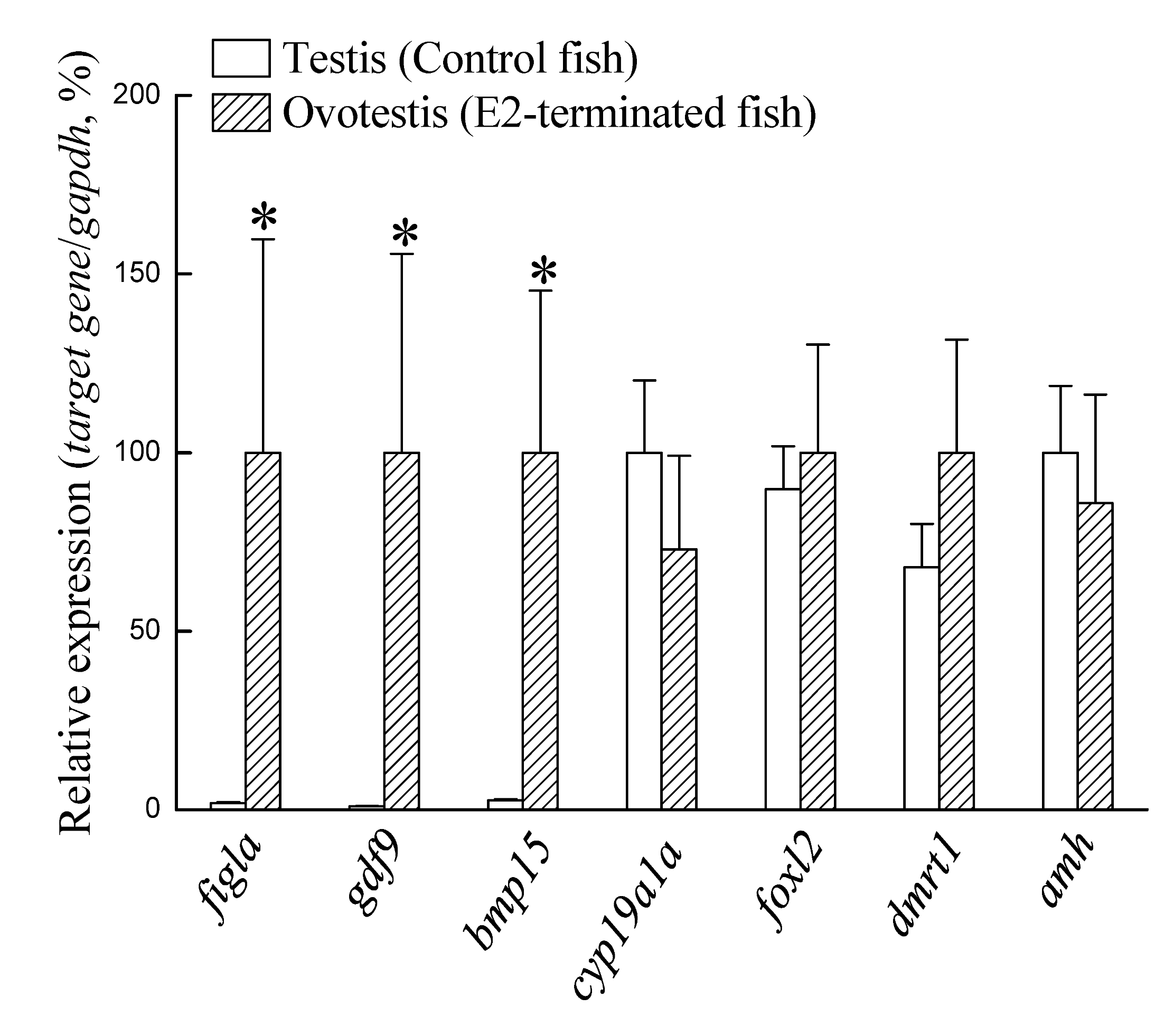

Supplement: S4 Fig — We created an ovotestis by ectopically inducing oocytes in the testicular region with estradiol (E2) administration and then E2 withdrawal. Normal testis (n = 8) and E2-induced ovotestis (n = 4) were used for RNA analysis. qPCR data confirmed that oocytes-expressed figla, gdf9, and bmp15 were expressed at higher levels in the ovotestes than in the testes. No difference of Sertoli cells marker (dmrt1 and amh) and follicle cells marker (foxl2 and cyp19a1a) were observed in the ovotestes than in the testes. An asterisk indicates a significant difference between the testes and ovotestis (P < 0.05). (TIFF) [file pone.0186991.s005.tiff]
